# Lysine methylation of PPP1CA by the methyltransferase SUV39H2 disrupts TFEB-dependent autophagy and promotes intervertebral disc degeneration

**DOI:** 10.1038/s41418-023-01210-4

**Published:** 2023-08-21

**Authors:** Huaizhen Liang, Rongjin Luo, Gaocai Li, Weifeng Zhang, Dingchao Zhu, Di Wu, Xingyu Zhou, Bide Tong, Bingjin Wang, Xiaobo Feng, Kun Wang, Yu Song, Cao Yang

**Affiliations:** 1 Department of Orthopaedics, Union Hospital, Tongji Medical College, Huazhong University of Science and Technology, Wuhan, 430022 China; 2 Department of Spine Surgery, Honghui Hospital, Xi’an Jiaotong University, Xi’an, 710054 China

**Keywords:** Rheumatic diseases, Macroautophagy

## Abstract

Impaired transcription factor EB (TFEB) function and deficient autophagy activity have been shown to aggravate intervertebral disc (IVD) degeneration (IDD), yet the underlying mechanisms remain less clear. Protein posttranslational modifications (PTMs) are critical for determining TFEB trafficking and transcriptional activity. Here, we demonstrate that TFEB activity is controlled by protein methylation in degenerated nucleus pulposus cells (NPCs), even though TFEB itself is incapable of undergoing methylation. Specifically, protein phosphatase 1 catalytic subunit alpha (PPP1CA), newly identified to dephosphorylate TFEB, contains a K141 mono-methylated site. In degenerated NPCs, increased K141-methylation of PPP1CA disrupts its interaction with TEFB and subsequently blocks TEFB dephosphorylation and nuclear translocation, which eventually leads to autophagy deficiency and NPC senescence. In addition, we found that the PPP1CA-mediated targeting of TFEB is facilitated by the protein phosphatase 1 regulatory subunit 9B (PPP1R9B), which binds with PPP1CA and is also manipulated by K141 methylation. Further proteomic analysis revealed that the protein lysine methyltransferase suppressor of variegation 3–9 homologue 2 (SUV39H2) is responsible for the K141 mono-methylation of PPP1CA. Targeting SUV39H2 effectively mitigates NPC senescence and IDD progression, providing a potential therapeutic strategy for IDD intervention.

## Introduction

Low back pain is one of the most prevalent conditions that greatly impacts quality of life. Intervertebral disc (IVD) degeneration (IDD) is a chronic degenerative disease that is strongly associated with low back pain [1]. As degeneration proceeds, nucleus pulposus cell (NPC) dysfunction and extracellular matrix remodeling occur in the IVD tissue [2, 3]. Cellular senescence is an alternative fate of NPCs and is characterized by stable cell cycle arrest and the upregulation of related markers such as cyclin-dependent kinase inhibitor 2 A (CDKN2A/p16) and senescence-associated β-galactosidase (SA-β-gal), which play an important role in the process of disc degeneration [4–6]. However, the underlying molecular mechanisms of senescence in NPCs remain poorly understood.

As an important player in the maintenance of cellular homeostasis, autophagy is a conserved cellular adaptive response for the clearance of damaged intracellular components through the lysosomal degradation pathway [7]. A decrease in autophagic activity is associated with NPC senescence and is implicated in IDD processes [8, 9]. Transcription factor EB (TFEB), a major regulator of autophagy, modulates autophagic activity at the transcriptional level by binding to the promoters of autophagy/lysosome-related genes [10–12]. TFEB senses multiple stress and nutrient fluctuations within the cell and shuttles to the nucleus to activate its downstream target genes, a process that largely depends on its altered phosphorylation status [13]. Impaired nuclear localization of TFEB has been implicated in various degenerative diseases, including IDD [14, 15]. Previous studies have provided evidence that TFEB activity is decreased in nucleus pulposus (NP) tissues and that the recovery of TFEB activity effectively restores autophagic activity to inhibit NPC senescence and IDD progression [16, 17]. However, the precise mechanisms by which TFEB activity is regulated in IDD progression remain unclear.

TFEB activity is modulated by a range of posttranslational modifications (PTMs), including phosphorylation, ubiquitination, PARsylation, sulfhydration and alkylation [13, 18–21]. As a major protein PTM, the role of lysine methylation in histone regulation is well characterized. Recently, increasing evidence has suggested that lysine methylation is involved in non-histone-associated signal transduction as a novel regulatory mechanism [22]. However, whether lysine methylation is involved in TFEB activation in IDD progression remains unknown.

Here, we report for the first time the vital role of lysine methylation in TFEB regulation during IDD and NPC senescence. We provide evidence that the PPP1CA/PPP1R9B phosphatase complex is a novel regulator of TFEB and is responsible for its dephosphorylation and subsequent activation. Further proteomic analysis revealed that the methyltransferase SUV39H2 interacts with and methylates PPP1CA, prevents it from binding to TFEB, and leads to TFEB cytoplasmic retention and impaired autophagy. Our findings reveal crosstalk between methylation and phosphorylation involving TFEB regulation, and SUV39H2 could be a potential target for the treatment of IDD.

## Results

### NPC senescence and impaired autophagic activity are strongly correlated with IDD progression

**Fig. 1:**
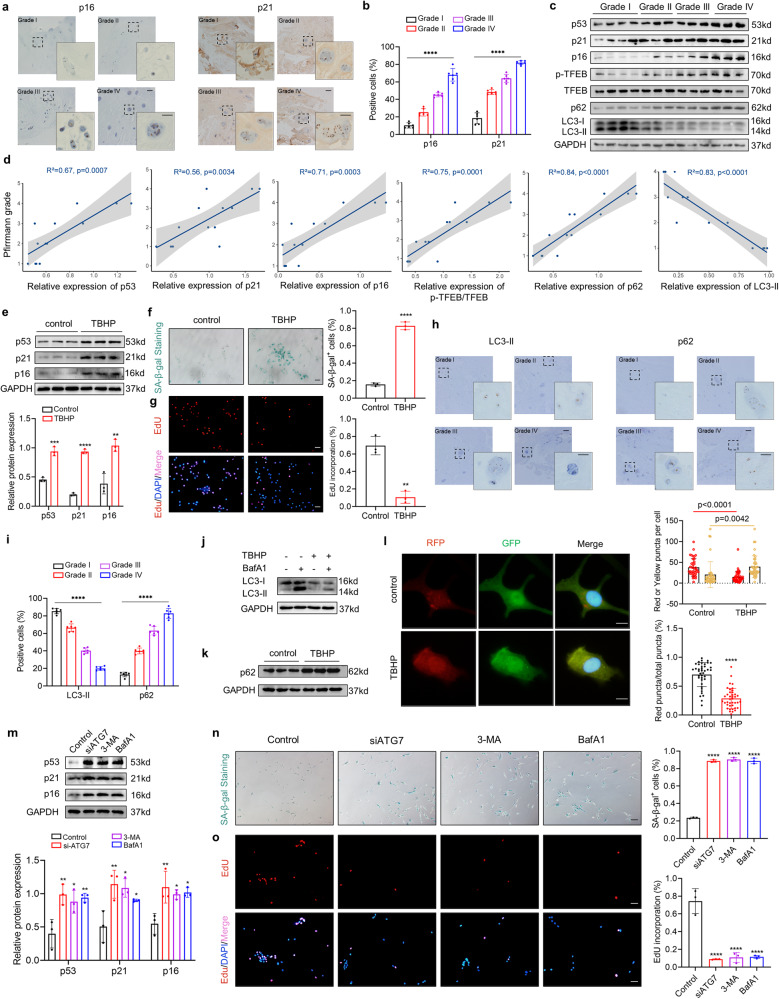
NPC senescence and impaired autophagic activity are strongly correlated with IDD progression. **a** IHC staining of p16 and p21 in human NP tissues (Scale bar: 100 μm, 50 μm). **b** Comparison of the number of p16/p21 positive cells in human NP tissues. **c** Western blot analysis of p53, p21, p16, TFEB, p-TFEB, p62, LC3-II in human NP tissues. **d**) Linear regression analysis between the p53, p21, p16, TFEB, p-TFEB, p62, LC3-II protein levels and the Pfirrmann MRI grades. **e** Western blot analysis of p53, p21, p16 in human NPCs treated with 50 μM TBHP for 24 h. **f** SA‐β‐gal activity staining and analysis of NPCs with the indicated treatment (Scale bar: 100 μm). **g** EdU incorporation assay and analysis of NPCs with the indicated treatment (Scale bar: 100 μm). **h** IHC staining of p62 and LC3-II in human NP tissues (Scale bar: 100 μm, 50 μm). **i** Comparison of the number of p62/LC3-II positive cells in human NP tissues. **j, k** Western blot analysis of LC3-II and p62 in human NPCs with the indicated treatment. **l** Representative images of stubRFP-sensGFP-LC3 puncta in NPCs and number of autophagosomes (yellow dots) and autolysosomes (red dots) per cell and ratio of red: total puncta in NPCs with the indicated treatment (Scale bar: 10 μm). **m** Western blot analysis of p53, p21, p16 in human NPCs with the indicated treatment. **n** SA‐β‐gal activity staining and analysis of NPCs with the indicated treatment (Scale bar: 100 μm). **o** EdU incorporation assay and analysis of NPCs with the indicated treatment (Scale bar: 100 μm). Data are expressed as mean ± SD. ^*^p < 0.05, ^**^p < 0.01, ^**^p < 0.001, ^****^p < 0.0001, ns not significant, two-tailed unpaired *t*-test.

To dissect the role of cellular senescence in IDD progression, we collected intervertebral disc tissue samples from patients. Based on the Pfirrmann MRI classification system, healthy or differently degenerated disc tissues were classified as grade I/II/III/IV. We first analyzed the expression of senescence-associated markers in NP tissues, and these markers were dramatically elevated in degenerated NP tissues (Fig. 1a–c). In addition, linear regression analysis of senescence-associated protein expression levels and IVD degeneration grade showed that senescence-associated markers levels were positively correlated with IVD degeneration grade (Fig. 1d). Furthermore, we developed an in vitro model of NPC degeneration by exposing NPCs to tert-butyl-hydroperoxide (TBHP) based on a previous study [16]. TBHP-treated NPCs consistently exhibited multiple senescence phenotypes, including upregulated expression of senescence-associated markers, a decreased ratio of EdU-positive cells, and increased SA-β-Gal activity (Fig. 1e–g). Autophagy is a regulatory mechanism of cellular homeostasis, and decreased autophagy has been implicated in a variety of degenerative diseases. Indeed, impaired autophagy in IDD tissues was evidenced by reduced LC3-II expression and elevated p62 expression, as indicated by immunohistochemistry (IHC) analysis and immunoblot analysis (Fig. 1c, d, h, i). Subsequently, we determined the autophagic activity of TBHP-treated NPCs by LC3-II autophagic flux. Reduced LC3-II flux was observed in NPCs after TBHP treatment, as shown in Fig. 1j. Moreover, the expression of the autophagic substrate p62 was elevated in NPCs after TBHP treatment (Fig. 1k, Fig. S1b). To further confirm the above findings, NPCs transduced with stubRFP-sensGFP-LC3 were used to monitor autophagosome maturation. The percentage of red-only puncta, representing autolysosomes, was significantly decreased in TBHP-treated NPCs (Fig. 1l). To investigate whether autophagy mediates NPC senescence, we performed senescence analysis on NPCs after knockdown of ATG7 or treatment with two autophagy inhibitors, 3-MA or BafA1. The protein levels of senescence-related markers were elevated strongly in NPCs treated with si-ATG7, 3-MA or BafA1 (Fig. 1m). Consistently, SA-β-Gal staining and EdU incorporation assay indicated that autophagy blockade could accelerate NPC senescence (Fig. 1n, o). This line of evidence suggests that NPC senescence and impaired autophagic activity are strongly correlated with IDD progression and decreased autophagic activity contributes to increased senescence in NPCs in IDD.

### Impaired TFEB-regulated lysosomal dysregulation contributes to NPC senescence

**Fig. 2:**
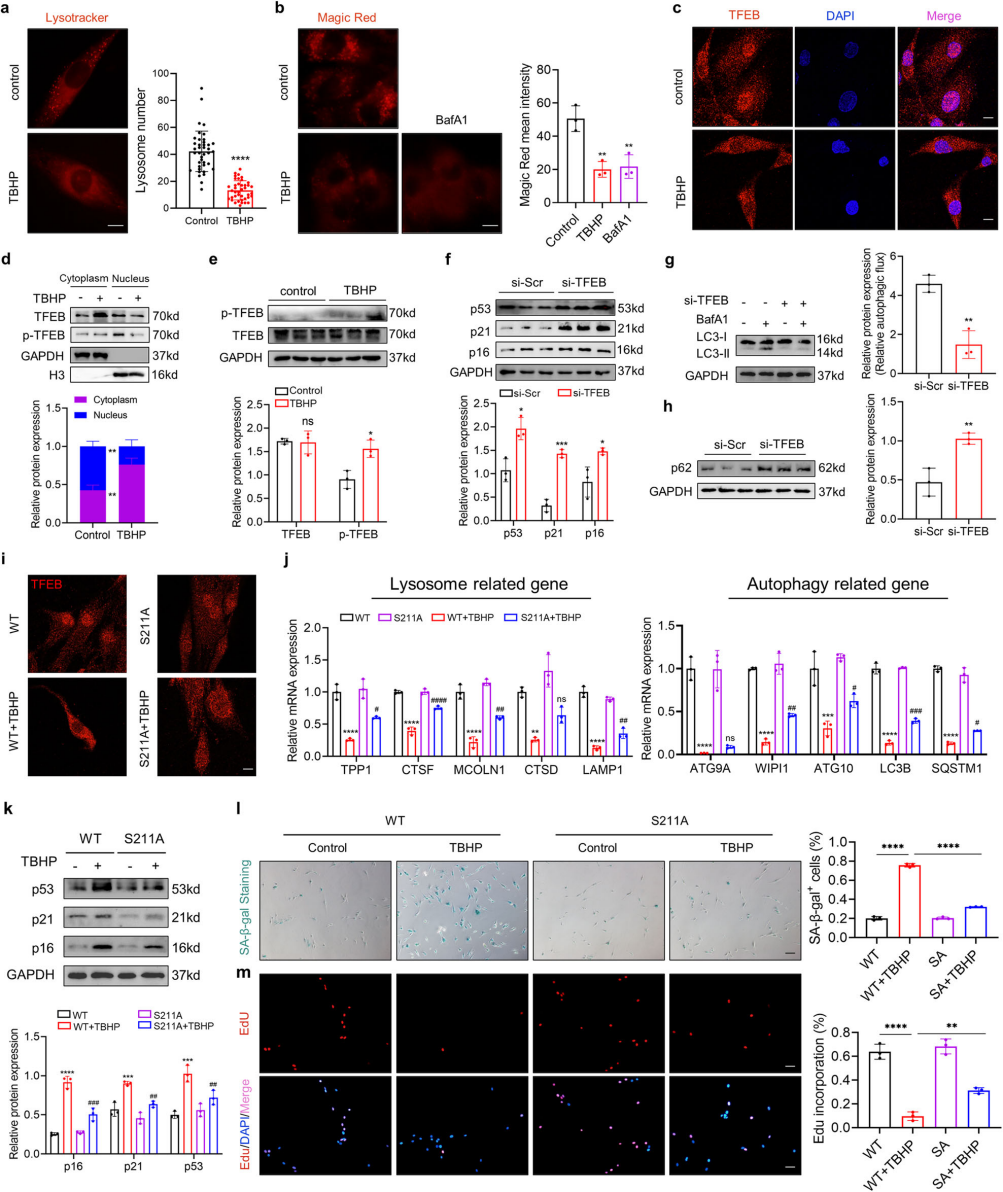
Impaired TFEB-regulated lysosomal dysregulation contributes to NPC senescence. **a** Representative images and quantification of total LysoTracker Red in NPCs with the indicated treatment (Scale bar: 10 μm). **b** Representative images and quantification of Magic Red dye in NPCs with the indicated treatment (Scale bar: 10 μm). **c** IF staining of TFEB in NPCs with the indicated treatment and the quantification of TFEB expression in the nucleus labeled with DAPI (Scale bar: 10 μm). **d** Cellular fractionation analysis of TFEB in NPCs with the indicated treatment. **e** Western blot analysis of p-TFEB and TFEB in human NPCs with the indicated treatment. **f** Western blot analysis of p53, p21, p16 in human NPCs transfected with si-TFEB. **g, h** Western blot analysis of LC3/p62 in human NPCs transfected with si-TFEB. **i** IF staining of TFEB in NPCs with the indicated treatment (Scale bar: 10 μm). **j** RT-qPCR analysis for TFEB target genes (TPP1, CTSF, MCOLN1, CTSD, LAMP1, ATG9A, WIPI1, ATG10, LC3B, SQSTM1) in NPCs with the indicated treatment. **k** Western blot analysis of p53, p21, p16 in human NPCs with the indicated treatment. **l** SA‐β‐gal activity staining and analysis of NPCs with the indicated treatment (Scale bar: 100 μm). **m** EdU incorporation assay and analysis of NPCs with the indicated treatment (Scale bar: 100 μm). Data are expressed as mean ± SD. ^*^p < 0.05, ^**^p < 0.01, ^**^p < 0.001, ^****^p < 0.0001, ns not significant, two-tailed unpaired *t-*test and and one-way ANOVA.

Completion of the autophagic process depends on the fusion of newly synthesized autophagosomes with lysosomes, while lysosomal dysfunction could impede the clearance of autophagosomes. We next evaluated the status of lysosomes in degenerated NPCs. We found that TBHP treatment reduced the number of lysosomes, as indicated by LysoTracker staining (Fig. 2a). Further, we used Magic Red to assess the proteolytic activity of lysosomes. The results showed that the red fluorescence intensity of TBHP-treated NPC was comparable to that of BafA1, which is a lysosomal acidification inhibitor and is used here as a positive control (Fig. 2b). To investigate whether TBHP treatment led to lysosomal membrane damage, we used immunofluorescence assays to assess whether the lysosomal marker enzyme cathepsin B leaks into the cytoplasm. The results shown that the intensity of cathepsin B and LAMP2 colocalization was similar between control and TBHP-treated cell. However, LLOMe exposure (positive control) induced significant cathepsin B cytosolic release which indicated by a reduced colocalization between cathepsin B and LAMP2 (Fig. S2a). This suggests that lysosomal stress levels are lower in our model. Degenerated NPCs are accompanied by autophagic failure and lysosomal dysregulation, which are transcriptionally controlled by a master regulator, TFEB. Next, we evaluated TFEB activity in degenerated NPCs. Under TBHP stimulation, TFEB mRNA and protein expression did not change significantly (Fig. S2b, c). However, TFEB nuclear localization was decreased in degenerated NPCs (Fig. 2c, d). Moreover, the phosphorylation level of TFEB, which determines its nuclear translocation, was significantly elevated in degenerated NPCs (Fig. 2e). To assess whether the loss of TFEB activity is associated with any compensatory activation of other TFE/MITF family of transcriptional factors, we evaluated the status of o MITF and TFE3. The results showed that the nuclear translocation of MITF and TFE3 was similar between control and TBHP-treated cell (Fig. S2d, e). Previous studies have shown that mTOR is the major phosphokinase complex regulating TFEB phosphorylation. However, immunofluorescence staining showed that TBHP treatment significantly reduced the nuclear translocation of TFEB in NPC treated with Torin1, a strong mTOR inhibitor, suggesting that TBHP treatment-induced TFEB hyperphosphorylation is due to a regulatory mechanism independent of mTOR (Fig. S2f). The ubiquitin-proteasome pathway is also involved in the regulation of TFEB activity through degrading phosphorylated TFEB. We used MG132, a proteasome inhibitor, to clarify whether the ubiquitin proteasome pathway is involved in the TBHP-induced cytoplasmic retention of TFEB. The results showed that TBHP treatment significantly increased TFEB phosphorylation levels and enhanced its binding with 14-3-3, even when the proteasome pathway was inhibited (Fig. S2g, h). These suggest that TBHP-induced cytoplasmic accumulation of TFEB is not dependent on the proteasome pathway. To further explore the role of TFEB in NPC senescence, we first knocked down TFEB in NPCs with an siRNA (Fig. S2i, j). Immunoblot analysis showed that TFEB knockdown strongly induced impaired autophagy and cellular senescence (Fig. 2f–h). Further, we used the TFEB S211A mutant to sort out the link between TFEB phosphorylation and NPC senescence. We re-expressed wild-type TFEB and S211A mutant in TFEB-depleted NPCs. RT‒qPCR analysis and immunofluorescence analysis showed that the reconstituted expression of S211A mutant in NPCs blocked TBHP-reduced TFEB inactivation (Fig. 2i, j). Importantly, TFEB S211A mutant could rescue the senescence phenotype in TBHP-treated NPCs, as indicated by western blot, SA-β-gal activity analysis and EdU incorporation assay (Fig. 2k–m). Taken together, these results indicated that impaired TFEB-regulated lysosomal dysregulation and autophagic failure contributes to NPC senescence.

### Lysine methylation is involved in TFEB inactivation and IDD progression

**Fig. 3:**
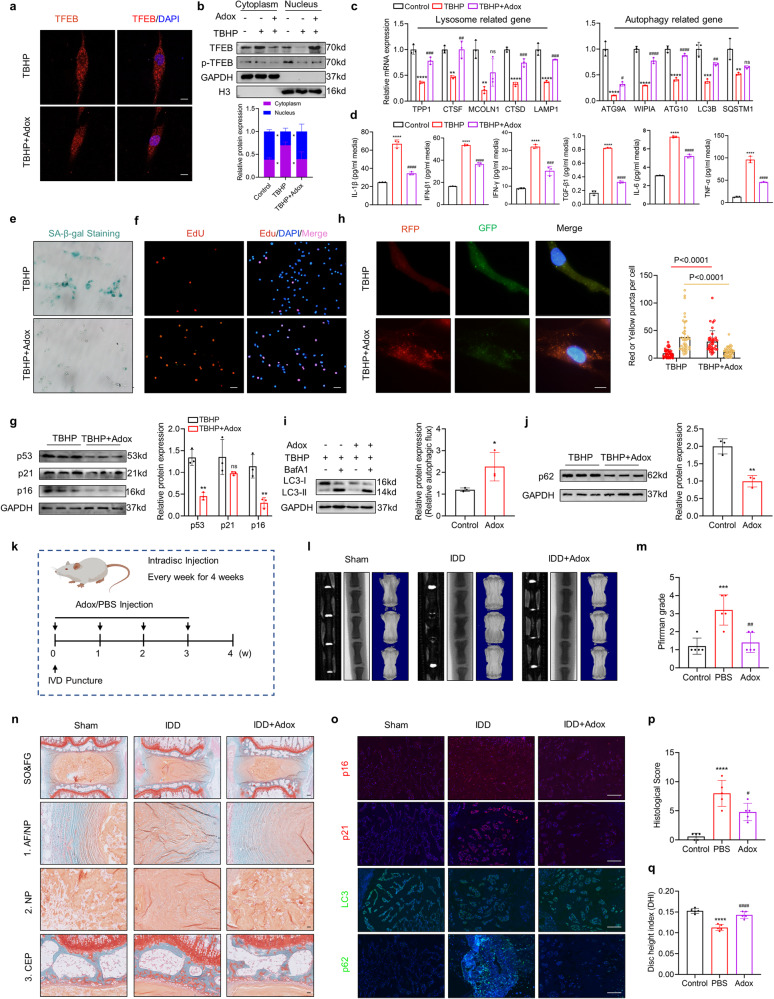
Lysine methylation is involved in TFEB inactivation and IDD progression. **a** IF staining of TFEB in NPCs treat with 100 μM Adox and the quantification of TFEB expression in the nucleus labeled with DAPI (Scale bar: 10 μm). **b** Cellular fractionation analysis of TFEB in NPCs with the indicated treatment. **c** RT-qPCR analysis for TFEB target genes (TPP1, CTSF, MCOLN1, CTSD, LAMP1, ATG9A, WIPI1, ATG10, LC3B, SQSTM1) in NPCs with the indicated treatment. **d** ELISA analysis for autophagy-related cytokines (IL-1β, IFN-β1, IFN-γ, TGF-β1, IL-6, TNF-α) in NPCs with the indicated treatment. **e** SA‐β‐gal activity staining and analysis of NPCs with the indicated treatment (Scale bar: 100 μm). **f** EdU incorporation assay and analysis of NPCs with the indicated treatment (Scale bar: 100 μm). **g** Western blot analysis of p53, p21, p16 in human NPCs with the indicated treatment. **h** Representative images of stubRFP-sensGFP-LC3 puncta and number of autophagosomes (yellow dots) and autolysosomes (red dots) per cell (Scale bar: 10 μm). **i, j** Western blot analysis of LC3 and p62 in human NPCs with the indicated treatment. **k** Schematic illustration of the experiment design. **l** Representative X-ray, micro-CT, MRI images of coccygeal vertebrae from rats with the indicated treatment. **m** The Pfirrmann MRI grade scores of coccygeal vertebrae from rats with the indicated treatment. (*n*  =  5). **n** SO&FG staining of coccygeal vertebrae from rats with the indicated treatment (Scale bar: 200 μm, 50 μm). **o** IF staining of p16, p21, LC3, p62 in coccygeal IVDs from rats with the indicated treatment (Scale bar: 100 μm). Histological score (**p**) and Disc height index (**q**) of coccygeal IVDs from rats with the indicated treatment. Data are expressed as mean ± SD. ^*^p < 0.05, ^**^p < 0.01, ^***^p < 0.001, ^****^p < 0.0001, ns not significant, two-tailed unpaired *t* test (**e–j**) and one-way ANOVA (**b–d, m, p, q**).

Activation of TFEB is regulated by a range of protein PTMs, including phosphorylation, ubiquitination, sulfhydration and alkylation. As an integral type of protein modification, lysine methylation is involved in both chromatin-associated and non-chromatin-associated signaling pathways, and these pathways play an important role in various aspects of cellular biology. However, the relationship between lysine methylation and TFEB and its role in IDD remain elusive. First, we assessed the effect of lysine methylation on TFEB activity by using the pan-methylation inhibitor Adox, and the results showed that Adox treatment rescued TBHP-reduced TFEB activity, as evidenced by the elevated nuclear accumulation of TFEB and the upregulated expression of its downstream gene (Fig. 3a–c). Subsequently, Adox treatment effectively alleviated the TBHP-induced increase in senescence-associated marker expression, delayed proliferation, and increased SA-β-Gal activity (Fig. 3d–g, Fig. S3a, b). In addition, Adox partially reversed the autophagic activity of NPCs treated with TBHP, as evidenced by the elevated autophagic flux (Fig. 3h–j and Fig. S3c). To further assess the role of lysine methylation in IDD progression in vivo, a needle puncture-induced IDD rat model was established (Fig. 3k) [23]. X-ray and micro-CT analyses suggested Adox treatment substantially attenuated the loss of disc height (Fig. 3l, q). Results from MRI examination revealed the T2-weighted signals of the IVD in rats treated with PBS were lower than those in rats received Adox injection (Fig. 3l, m). Moreover, H&E and SO&FG staining showed that Adox treatment significantly attenuated IVD histological degeneration, as evidenced by improved extracellular matrix arrangement, elevated NP tissue area and a clearer boundary between NP and AF (Fig. 3n, p and Fig. S3d). Importantly, the expression of senescence-related markers was significantly decreased after the intradiscal injection of Adox, while autophagic activity was restored (Fig. 3o and Fig. S3d). Collectively, these data indicate that reduced TFEB activity in IDD progression may be regulated by lysine methylation.

### K141 methylation of PPP1CA disrupts its binding with TFEB

**Fig. 4:**
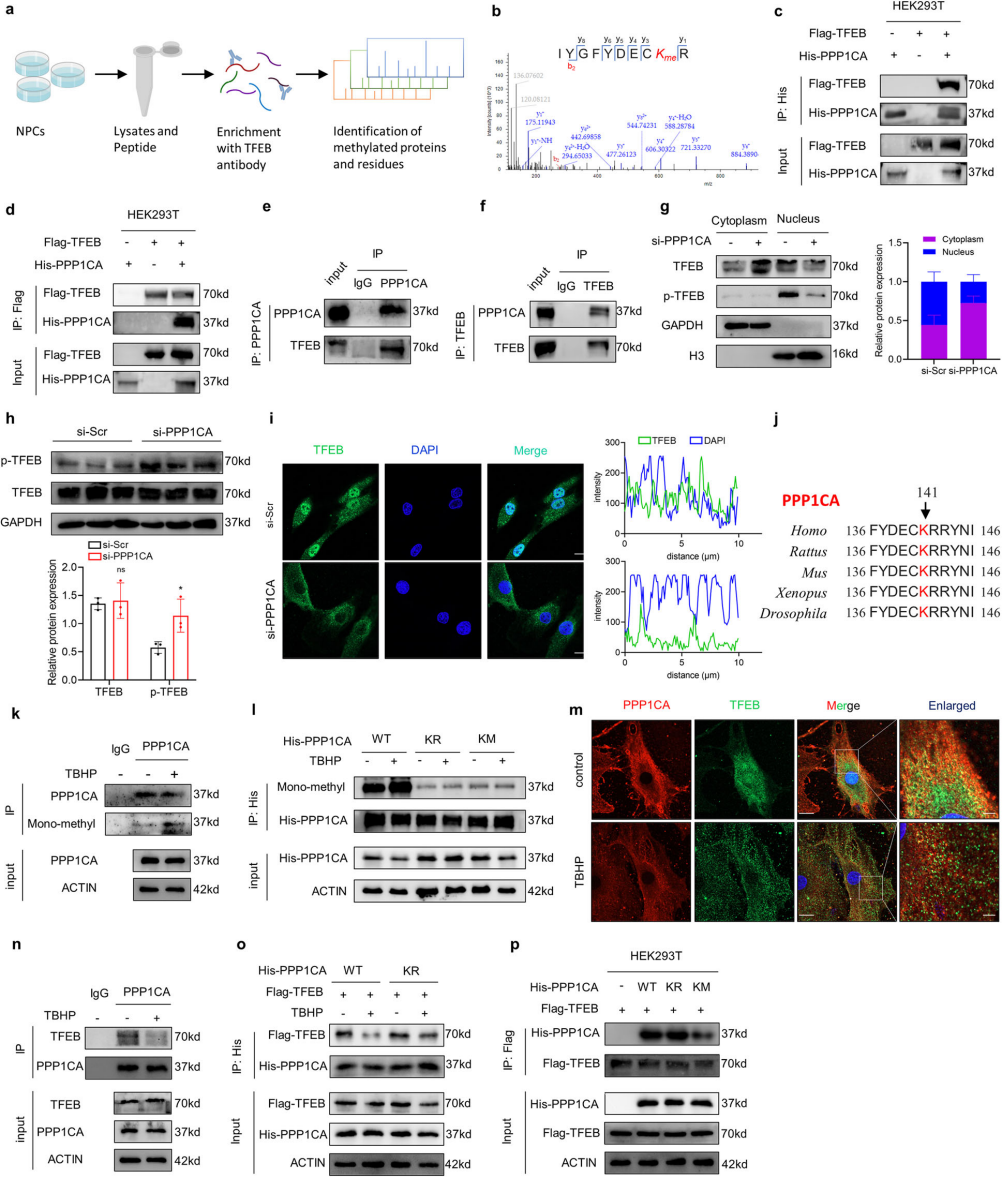
K141 methylation of PPP1CA disrupts its binding with TFEB. **a** Schematic workflow showing TFEB Co-IP with NPCs followed by LC‒MS/MS experiments. **b** LC-MS/MS spectrum of the tryptic peptide IYGFYDECKR, carrying a mass of +14.0156 Da at residues Lys141. **c, d** Exogenous Co-IP analysis of the interaction of TFEB with PP1 in HEK293T cells cotransfected with Flag-TFEB and His-PPP1CA. **e, f** Endogenous Co-IP analysis of the interaction of TFEB with PPP1CA in NPCs. **g** Cellular fractionation analysis of TFEB in NPCs transfected with si-PPP1CA. **h** Western blot analysis of p-TFEB and TFEB in human NPCs transfected with si-PPP1CA. (**i**) IF staining of TFEB in NPCs transfected with si-PPP1CA and the quantification of TFEB expression in the nucleus labeled with DAPI (Scale bar: 10 μm). **j** Sequence alignment of PPP1CA across multiple species near the K141. **k** Co-IP analysis of methylated PPP1CA in NPCs with the indicated treatment. **l** Co-IP analysis of methylated PPP1CA in NPCs transfected with PPP1CA His-tagged wild-type PPP1CA, KR mutants and KM mutants. **m** IF analysis of the interaction of TFEB with PPP1CA in NPCs treated with the indicated treatment (Scale bar: 10 μm, 2μm). **n** Co-IP analysis of the interaction of TFEB with PPP1CA in NPCs with the indicated treatment. **o** Co-IP analysis of the interaction of TFEB with PPP1CA in NPCs cotransfected with Flag-TFEB and His-PPP1CA (wild-type, KR mutants) with the indicated treatment. **p** Co-IP analysis of the interaction of TFEB with PPP1CA in HEK293T cells cotransfected with Flag-TFEB and His-PPP1CA (wild-type, KR mutants and KM mutants). Data are expressed as mean ± SD. ^*^p < 0.05, ns not significant, two-tailed unpaired *t* test.

To assess the methylation status of TFEB, we performed LC‒MS/MS in human NPCs (Fig. 4a). However, no TFEB methylation modification sites were found. Surprisingly, protein phosphatase PPP1CA, a TFEB-interacting protein, was found to possess a methylation modification site at lysine 141 (Fig. 4b). This led us to explore the relationship between PPP1CA and TFEB and the role that methylation plays in this relationship. First, an interaction between ectopically expressed TFEB and PPP1CA was observed by reciprocal coimmunoprecipitation (co-IP) in human embryonic kidney-293 T (HEK293T) cells (Fig. 4c, d), as well as between endogenous TFEB and PPP1CA in NPCs (Fig. 4e, f). Furthermore, the level of TFEB phosphorylation was significantly elevated in PPP1CA-knockdown NPCs and the nuclear localization of TFEB, which is mainly regulated by its phosphorylation level, was significantly decreased in NPCs with PPP1CA knockdown (Fig. 4g–i and Fig. S4a and S4b). Next, we evaluated the binding of TFEB to 14-3-3, which is regulated by the TFEB phosphorylation status. The results showed that PPP1CA knockdown enhanced TFEB binding to 14-3-3 (Fig. S4c). However, mTORC1 activity was not significantly altered in PPP1CA knockdown NPCs, as evidenced by the phosphorylation of mTORC1 substrates 4EBP1 and S6K (Fig. S4d). These data indicate that PPP1CA interacts with and dephosphorylates TFEB in an mTORC1 non-dependent manner. Next, we investigated whether lysine 141 of PPP1CA was methylated. Sequence alignment across multiple species demonstrated that lysine 141 of PPP1CA is an evolutionarily conserved residue (Fig. 4j). Using an anti-pan-methylation antibody, we detected methylation in endogenous PPP1CA immunoprecipitates, and PPP1CA methylation levels were elevated in NPCs treated with TBHP (Fig. 4k). Furthermore, we mutated lysine 141 of PPP1CA to arginine or methionine, where lysine to arginine (KR) was used as a methyl-deficient mutation and lysine to methionine (KM) was used as a methyl-mimetic mutation [24]. Strikingly, the methylation levels of both KR and KM mutants were significantly decreased, and TBHP treatment did not increase the methylation levels of the KR/KM mutants compared with those of wild-type PPP1CA (Fig. 4l). These data suggest that PPP1CA is methylated at lysine 141. Subsequently, we evaluated the colocalization of TFEB with PPP1CA and showed that the intensity of TFEB and PPP1CA colocalization was significantly decreased in TBHP-treated NPCs (Fig. 4m). The interaction between endogenous TFEB and PPP1CA was consistently weakened in NPCs treated with TBHP (Fig. 4n). The recruitment of TFEB to lysosomes is closely related to its phosphorylation status, so we next assessed whether TFEB binds to PPP1CA at the lysosome. The results seem to show some binding of PPP1CA/TFEB to lysosomes, however, TBHP treatment did not change their co-localization (Fig. S4e). To investigate whether the attenuated interaction between TFEB and PPP1CA was mediated by PPP1CA methylation, KR mutants were transfected into NPCs. Upon TBHP stimulation, the interaction between Flag-TFEB and wild-type PPP1CA but not the KR mutant was significantly reduced (Fig. 4o). Furthermore, we assessed the interaction between Flag-TFEB and His-PPP1CA in HEK293T cells. The results showed that the KM mutant but not the KR mutant or wild-type protein diminished the binding between Flag-TFEB and His-PPP1CA (Fig. 4p). Collectively, these data indicate that the K141 methylation of PPP1CA disrupts its binding with TFEB.

### K141 methylation of PPP1CA modulates TFEB, autophagic activity and cellular senescence in NPCs

**Fig. 5:**
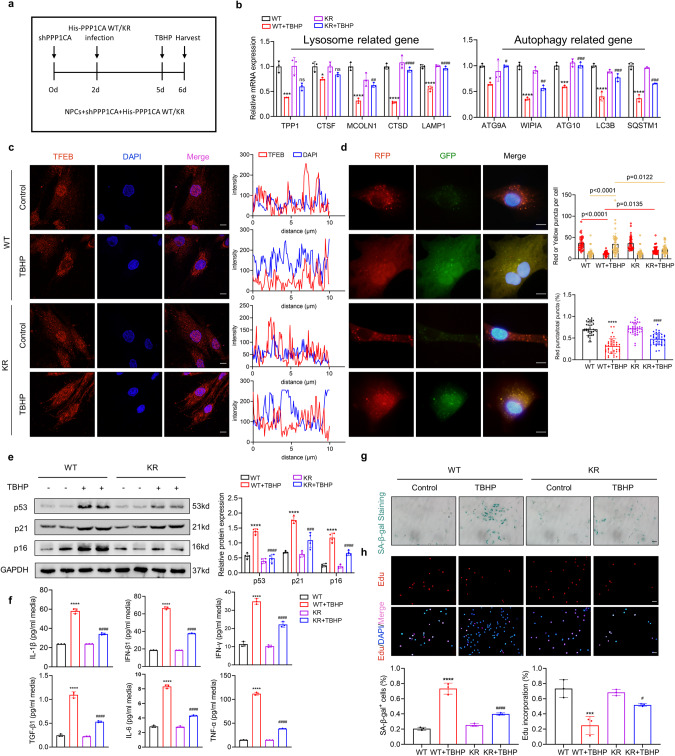
K141 methylation of PPP1CA modulates TFEB, autophagic activity and cellular senescence in NPCs. **a** Schematic illustration of the experiment design. **b** RT-qPCR analysis for TFEB target genes (TPP1, CTSF, MCOLN1, CTSD, LAMP1, ATG9A, WIPI1, ATG10, LC3B, SQSTM1) in NPCs reconstituted expression of wild-type PPP1CA or KR mutant with the indicated treatment. **c** IF staining of TFEB in NPCs reconstituted expression of wild-type PPP1CA or KR mutant with the indicated treatment (Scale bar: 10 μm). **d** Representative images of stubRFP-sensGFP-LC3 puncta and number of autophagosomes (yellow dots) and autolysosomes (red dots) per cell and ratio of red: total puncta in NPCs reconstituted expression of wild-type PPP1CA or KR mutant with the indicated treatment (Scale bar: 10 μm). **e** Western blot analysis of p53, p21, p16 in human NPCs reconstituted expression of wild-type PPP1CA or KR mutant with the indicated treatment. **f** ELISA analysis for autophagy-related cytokines (IL-1β, IFN-β1, IFN-γ, TGF-β1, IL-6, TNF-α) in NPCs reconstituted expression of wild-type PPP1CA or KR mutant with the indicated treatment. **g** SA‐β‐gal activity staining and analysis of NPCs reconstituted expression of wild-type PPP1CA or KR mutant with the indicated treatment (Scale bar: 100 μm). **h** EdU incorporation assay and analysis of NPCs reconstituted expression of wild-type PPP1CA or KR mutant with the indicated treatment (Scale bar: 100 μm). Data are expressed as mean ± SD. ^*^p < 0.05, ^**^p < 0.01, ^***^p < 0.001, ^****^p < 0.0001, ns not significant, and one-way ANOVA.

To investigate the role of PPP1CA K141 methylation in degenerated NPCs, wild-type PPP1CA or KR mutants were re-expressed in PPP1CA-depleted NPCs (Fig. 5a). RT‒qPCR analysis of TBHP-treated NPCs transduced with KR mutants revealed an attenuated reduction in TFEB downstream gene expression compared to wild-type PPP1CA-transduced NPCs (Fig. 5b). Immunofluorescence (IF) analysis consistently showed that TBHP treatment significantly attenuated TFEB nuclear accumulation in NPCs transduced with wild-type PPP1CA but not in NPCs transduced with KR mutants (Fig. 5c). These data indicate that reduced TFEB activity is regulated by the K141 methylation of PPP1CA. Next, autophagic flux analysis showed that the reconstituted expression of KR mutants in NPCs blocked TBHP-reduced autophagic flux, as evidenced by restored LC3-II accumulation and the percentage of red-only puncta (Fig. 5d, S5a). Furthermore, the TBHP-induced expression of senescence-associated markers was abrogated by the reconstituted expression of KR mutants (Fig. 5e). Moreover, NPCs transduced with KR mutants were resistant to the TBHP-induced expression of senescence-associated secretory phenotype (Fig. 5f). In addition, TBHP-induced elevated SA-β-Gal activity and delayed proliferation were significantly attenuated in NPCs transduced with KR mutants compared with those transduced with wild-type PPP1CA (Fig. 5g, h). Together, these data indicate that the K141 methylation of PPP1CA and its inhibitory effect on TFEB activity is a key mechanism underlying TBHP-induced TFEB inactivation and subsequent impaired autophagy and senescence.

### K141 methylation of PPP1CA disrupts PPP1CA/PPP1R9B holoenzyme assembly

**Fig. 6:**
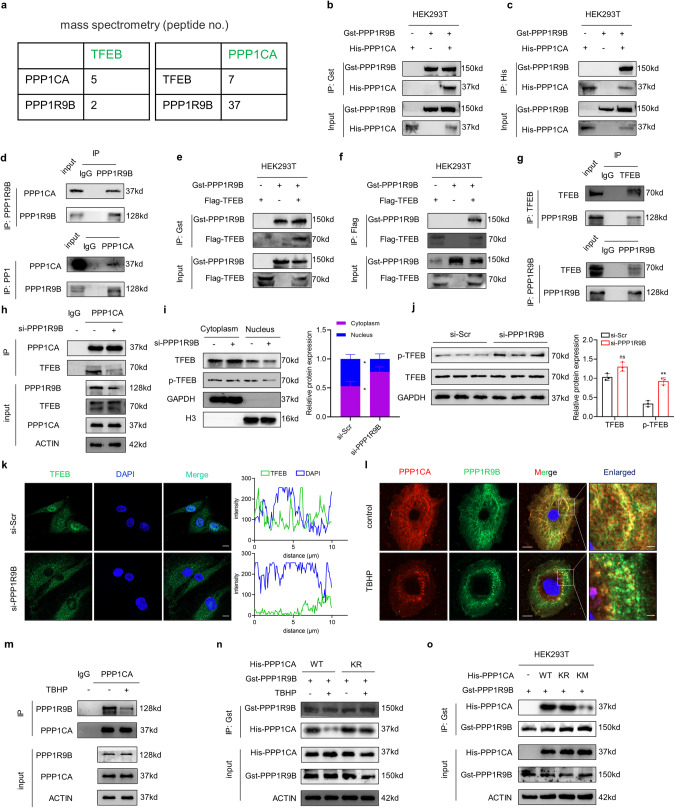
K141 methylation of PPP1CA disrupts PPP1CA/PPP1R9B holoenzyme assembly. **a** Mass spectrometry identification of peptide counts by TFEB or PPP1CA immunoprecipitation. **b, c** Exogenous Co-IP analysis of the interaction of PPP1R9B with PPP1CA in HEK293T cells cotransfected with Gst-PPP1R9B and His-PPP1CA. **d** Endogenous Co-IP analysis of the interaction of PPP1R9B with PPP1CA in NPCs. **e, f** Exogenous Co-IP analysis of the interaction of TFEB with PPP1R9B in HEK293T cells cotransfected with Flag-TFEB and Gst-PPP1R9B. **g** Endogenous Co-IP analysis of the interaction of TFEB with PPP1R9B in NPCs. **h** Co-IP analysis of the interaction of TFEB with PPP1CA in NPCs transfected with si-PPP1R9B. (**i**) Cellular fractionation analysis of TFEB in NPCs transfected with si-PPP1R9B. **j** Western blot analysis of p-TFEB and TFEB in human NPCs transfected with si-PPP1R9B. **k** IF staining of TFEB in NPCs transfected with si-PPP1R9B and the quantification of TFEB expression in the nucleus labeled with DAPI (Scale bar: 10 μm). **l** IF analysis of the interaction of PPP1R9B with PPP1CA in NPCs with the indicated treatment (Scale bar: 10 μm, 2 μm). **m** Co-IP analysis of the interaction of PPP1R9B with PPP1CA in NPCs with the indicated treatment. **n** Co-IP analysis of the interaction of PPP1R9B with PPP1CA in NPCs cotransfected with Gst-PPP1R9B and His-PPP1CA (wild-type, KR mutants) with the indicated treatment. **o** Co-IP analysis of the interaction of PPP1R9B with PPP1CA in HEK293T cells cotransfected with Gst-PPP1R9B and His-PPP1CA (wild-type, KR mutants and KM mutants). Data are expressed as mean ± SD. ^*^p < 0.05, ns not significant, two-tailed unpaired *t* test.

The PPP1CA holoenzyme consists of an activated catalytic subunit and a regulatory subunit. The substrate specificity of the PPP1CA catalytic core is dependent on the regulatory subunit [25, 26]. Interestingly, we identified a regulatory subunit, PPP1R9B, in the TFEB-interacting protein (Fig. 6a). To determine whether PPP1R9B targets PPP1CA to TFEB, we first evaluated the binding between PPP1CA, PPP1R9B, and TFEB. Co-IP assays showed ectopically expressed GST-PPP1R9B in the His-PPP1CA and Flag-TFEB immunoprecipitates and vice versa (Fig. 6b, c, e, f). Moreover, endogenous Co-IP results indicated that PPP1CA, PPP1R9B, and TFEB bind to each other (Fig. 6d, g). To further confirm the binding of TFEB, PPP1CA and TFEB, we immunoprecipitated PPP1CA from NPCs and followed by mass spectrometric analysis. We found that both PPP1R9B and TFEB are part of the PPP1CA interactome (Fig. 6a). In addition, knockdown of PPP1R9B in NPCs strongly disrupted the binding of PPP1CA to TFEB (Fig. 6h and Fig. S6a and S6b). We also observed a significant reduction in TFEB activity in NPCs with PPP1R9B knockdown, as indicated by reduced TFEB nuclear localization and elevated phosphorylation levels (Fig. 6i–k). These data suggest that the PPP1CA/PPP1R9B complex can bind to and dephosphorylate TFEB, with PPP1R9B playing a bridging role. Given that the PPP1CA targeting of TFEB is dependent on PPP1R9B and that the K141 methylation of PPP1CA blocks its binding to TFEB, we hypothesize that the K141 methylation of PPP1CA may disrupt the assembly of the PPP1CA/PPP1R9B complex. First, the binding of PPP1CA to PPP1R9B was assessed in TBHP-treated NPCs, and their interaction was significantly weakened (Fig. 6l, m). Then, we further mutated lysine 141 to arginine or methionine. Strikingly, in contrast to wild-type PPP1CA, the KR mutants displayed a negligible response in their binding with PPP1R9B upon TBHP treatment (Fig. 6n). In addition, the KM mutants but not the KR mutants or wild-type PPP1CA exhibited a significant reduction in binding to PPP1R9B (Fig. 6o). Collectively, these findings suggest that the K141 methylation of PPP1CA may disrupt PPP1CA/PPP1R9B holoenzyme assembly and subsequent TFEB activation.

### K141 methylation of PPP1CA is meditated by the methyltransferase SUV39H2

**Fig. 7:**
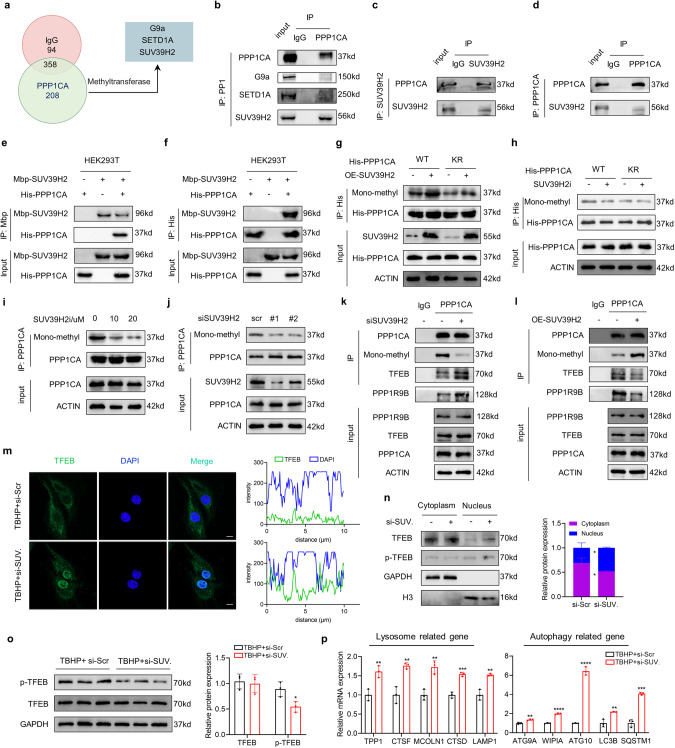
K141 methylation of PPP1CA is meditated by the methyltransferase SUV39H2. **a** LC‒MS/MS experiments was performed to identify three methyltransferases interacting with PP1 in NPCs. **b** Co-IP analysis of the interaction of PPP1CA with SUV39H2, SETD1A or G9a in NPCs. **c, d** Endogenous Co-IP analysis of the interaction of SUV39H2 with PPP1CA in NPCs. **e, f** Exogenous Co-IP analysis of the interaction of SUV39H2 with PPP1CA in HEK293T cells cotransfected with Mbp-SUV39H2 and His-PPP1CA. **g** Co-IP analysis of methylated PPP1CA in NPCs transfected with PPP1CA His-tagged wild-type PPP1CA, KR mutants and KM mutants with or without SUV39H2 overexpression. **h** Exogenous Co-IP analysis of methylated PPP1CA in NPCs transfected with PP1 His-tagged wild-type PPP1CA, KR mutants and KM mutants with or without 20 μM SUV39H2 inhibitor (SUV39H2i) treatment. **i** Endogenous Co-IP analysis of methylated PPP1CA in NPCs with indicated treatment. **j** Endogenous Co-IP analysis of methylated PPP1CA in NPCs transfected with si-SUV39H2. **k** Co-IP analysis of the interaction of PPP1CA with TFEB or PPP1R9B in NPCs transfected with si-SUV39H2. **l** Co-IP analysis of the interaction of PPP1CA with TFEB or PPP1R9B in NPCs SUV39H2 overexpression. **m** IF staining of TFEB in NPCs transfected with si-SUV39H2 and the quantification of TFEB expression in the nucleus labeled with DAPI (Scale bar: 10 μm). **n** Cellular fractionation analysis of TFEB in NPCs transfected with si-SUV39H2. **o** Western blot analysis of p-TFEB and TFEB in human NPCs transfected with si-SUV39H2. **p** RT-qPCR analysis for TFEB target genes (TPP1, CTSF, MCOLN1, CTSD, LAMP1, ATG9A, WIPI1, ATG10, LC3B, SQSTM1) in NPCs transfected with si-SUV39H2. Data are expressed as mean ± SD. ^*^p < 0.05, ^**^p < 0.01, ^***^p < 0.001, ns not significant, two-tailed unpaired *t* test.

To identify the upstream methyltransferase responsible for PPP1CA K141 methylation, we immunoprecipitated PPP1CA from NPCs and subsequently used LC–MS/MS to determine the PPP1CA-binding proteins. Three methyltransferases were identified: G9a, STED1A and SUV39H2 (Fig. 7a and Fig. S7a). Further Co-IP assays indicated that PPP1CA binds to SUV39H2 but not to G9a or SETD1A (Fig. 7b). This finding suggests that SUV39H2 potentially methylates PPP1CA. We detected an interaction between endogenous PPP1CA and SUV39H2 in NPCs (Fig. 7c, d). Subsequently, we also identified a relatively strong interaction between exogenous His-PPP1CA and Mbp-SUV39H2 in HEK293T cells (Fig. 7e, f). We also immunoprecipitated SUV39H2 followed by mass spectrometry to further characterize the interaction of SUV39H2 with PPP1CA. The mass spectrometry results once again demonstrated that the binding of SUV39H2 to PPP1CA (Fig. S7b). These results suggest that SUV39H2 is a PPP1CA-interacting protein. Next, we assessed whether SUV39H2 methylated K141 of PPP1CA. We overexpressed SUV39H2 in HEK293T cells and used the pan-lysine methylation antibody to detect the methylation of ectopically expressed PPP1CA. The results indicated that the methylation level of wild-type PPP1CA but not the KR mutant was increased in the cells with SUV39H2 overexpression (Fig. 7g). In addition, treatment with an inhibitor specific to SUV39H2 (SUV39H2i) strongly reduced the methylation of wild-type PPP1CA but not the KR mutant compared to the control groups (Fig. 7h). Importantly, SUV39H2i treatment or the depletion of SUV39H2 led to a significant reduction in endogenous PPP1CA methylation (Fig. 7i, j and Fig. S7c and S7d). These data suggest that SUV39H2 acts as a methyltransferase and is potentially responsible for methylating K141 of PPP1CA. Furthermore, we assessed the effect of SUV39H2 on the PPP1CA/PPP1R9B/TFEB complex. SUV39H2 knockdown enhanced the interaction of PPP1CA with PPP1R9B or TFEB, while SUV39H2 overexpression produced the opposite result (Fig. 7k, l). Moreover, TBHP treatment-induced TFEB activity reduction was restored by SUV39H2 depletion (Fig. 7m–p). Collectively, these findings suggest that SUV39H2 methylates K141 of PPP1CA and suppresses its phosphatase activity targeting TFEB.

### Suppression of SUV39H2 delays NPC senescence and IDD development

**Fig. 8:**
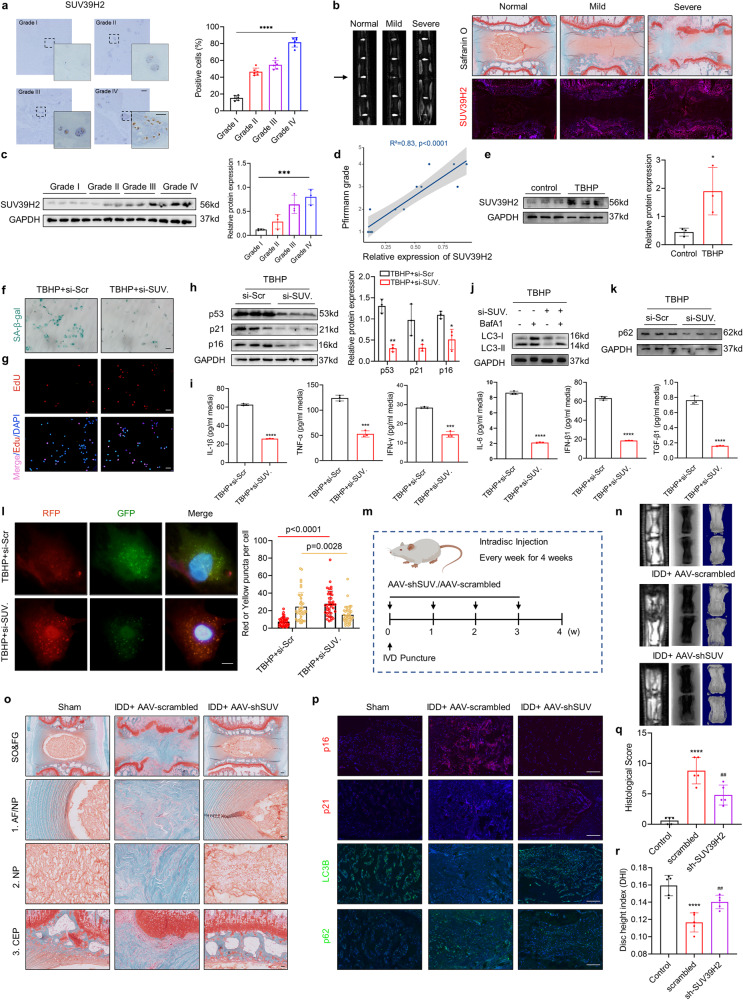
Suppression of SUV39H2 delays NPC senescence and IDD development. **a** IHC staining of SUV39H2 in human NP tissues. (Scale bar: 100 μm, 50 μm). **b** Representative MRI images and safranin O/fast green staining (top) and IF of SUV39H2 (bottom) in normal, moderate and severe degenerative IVDs (Scale bar: 200 μm). (**c**) Western blot analysis of SUV39H2 in human NP tissues. **d** Linear regression analysis between the SUV39H2 protein levels and the Pfirrmann MRI grades. **e** Western blot analysis of SUV39H2 in human NPCs with the indicated treatment. **f** SA‐β‐gal activity staining of NPCs transfected with si-SUV39H2. (Scale bar: 100 μm). **g** EdU incorporation assay of NPCs transfected with si-SUV39H2. (Scale bar: 100 μm). **h** Western blot analysis of p53, p21, p16 in human NPCs transfected with si-SUV39H2. **i** ELISA analysis for autophagy-related cytokines (IL-1β, IFN-β1, IFN-γ, TGF-β1, IL-6, TNF-α) in human NPCs transfected with si-SUV39H2. **j, k** Western blot analysis of LC3 and p62 in human NPCs transfected with si-SUV39H2. **l** Representative images of stubRFP-sensGFP-LC3 puncta in NPCs and number of autophagosomes (yellow dots) and autolysosomes (red dots) per cell in NPCs transfected with si-SUV39H2 (Scale bar: 10 μm). **m** Schematic illustration of the experiment design. **n** Representative X-ray, micro-CT, MRI images of coccygeal vertebrae from rats with the indicated treatment. **o** SO&FG staining of coccygeal vertebrae from rats with the indicated treatment (Scale bar: 200 μm, 50 μm). **p** IF staining of p16, p21, LC3, p62 in coccygeal IVDs from rats with the indicated treatment (Scale bar: 100 μm). Histological score (**q**) and Disc height index (**r**) of coccygeal IVDs from rats with the indicated treatment. Data are expressed as mean ± SD. ^*^p < 0.05, ^**^p < 0.01, ^****^p < 0.0001, ns not significant, two-tailed unpaired *t* test.

Given the regulation of the PPP1CA/PPP1R9B/TFEB complex by SUV39H2, we further investigated whether SUV39H2 expression changes during IDD progression. IHC analysis showed that SUV39H2 was elevated in patients with IDD (Fig. 8a). Furthermore, IF staining indicated that SUV39H2 expression increased as IVD tissue damage worsened in the needle puncture IDD rat model (Fig. 8b and Fig. S8a). Western blot analysis showed that SUV39H2 expression was elevated in degenerated NP tissues (Fig. 8c). Further linear regression analysis showed that SUV39H2 expression levels were positively correlated with IVD degeneration grade and autophagic failure (Fig. 8d and Fig. S8b, c). Additionally, TBHP-treated NPCs consistently displayed a marked increase in SUV39H2 (Fig. 8e). Collectively, these data demonstrate that SUV39H2 is increased in the NP of patients and rats with IDD, implicating a potential role for SUV39H2 in IDD progression. To further explore the role of SUV39H2 in NPC senescence as well as in IDD, we knocked down SUV39H2 in NPCs with an siRNA. NPCs with silenced SUV39H2 expression exhibited a significantly alleviated senescence-related phenotype compared with wild-type NPCs treated with TBHP, as indicated by decreased SA-β-gal activity and an increased ratio of EdU-positive cells (Fig. 8f, g and Fig. S8d and S8e). ELISA analysis and immunoblot analysis demonstrated that the expression of senescence-related markers was also decreased in SUV39H2-knockdown NPCs (Fig. 8h, i). Moreover, SUV39H2 depletion effectively alleviated TBHP-induced autophagic flux blockage (Fig. 8j–l and Fig. S8f–h). Collectively, these observations indicate that SUV39H2 knockdown can reverse impaired autophagy activity and cellular senescence in degenerated NPCs. Given that autophagy is negatively regulated by SUV39H2, we next examined whether the elevated SUV39H2 expression in degenerating NPCs was caused by autophagy decay. The results showed that the expression of SUV39H2 was not significantly altered (Fig. S8i). Next, we used adeno-associated virus (AAV) vectors to target and inhibit SUV39H2 in vivo. We injected AAV vectors carrying a short hairpin RNA targeting SUV39H2 into the NP region of IDD rats every week for four weeks (Fig. 8m). Strikingly, SUV39H2 knockdown significantly retarded the degeneration of IVD, as manifested by radiographic imaging and histological assessments (Fig. 8n, o, q, r and Fig. S8j and S8k). Importantly, the AAV-shSUV39H2-treated group exhibited enhanced autophagic activity and reduced expression of senescence-associated markers (Fig. 8p and Fig. S8j). Together, these findings suggest that the targeted inhibition of SUV39H2 could delay IDD progression.

## Discussion

**Fig. 9:**
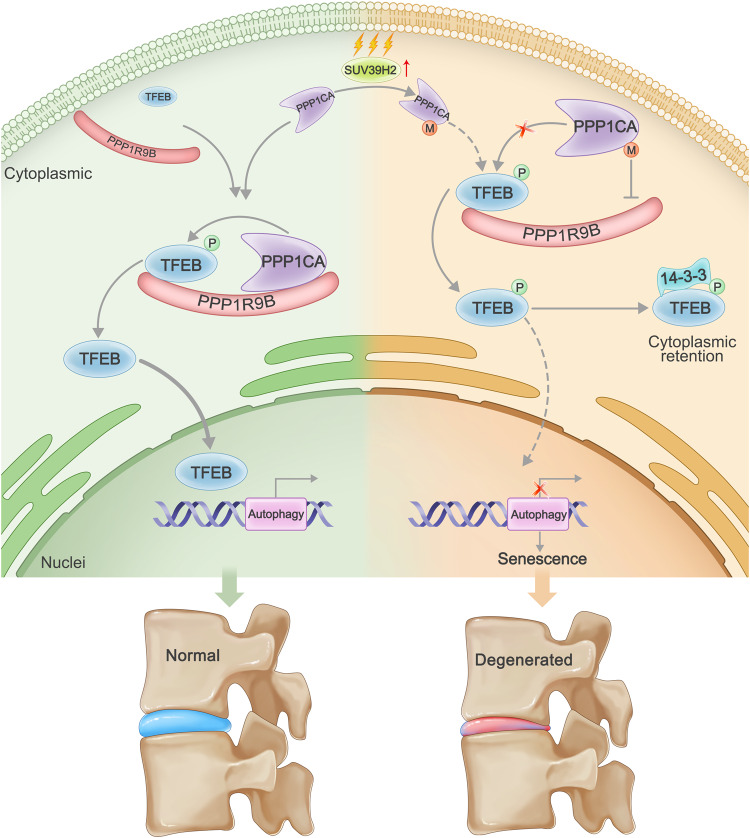
Schematic illustration of the mechanisms via which lysine methylation of PPP1CA by SUV39H2 promotes NPC senescence and IDD progression. In normal NPCs, PPP1CA/PPP1R9B phosphatase complex binds and dephosphorylates TFEB to maintain proper autophagic activity. In degenerated NPCs, upregulated SUV39H2 interacts with and methylates lysine 141 of PPP1CA. K141 methylation of PPP1CA weakens the phosphatase activity of the PPP1CA/PPP1R9B complex that binds to TFEB, which results in decreased TFEB-regulated autophagy activity and NPC senescence.

In this study, we first report that lysine methylation and the methyltransferase SUV39H2 actively participate in IDD progression through TFEB-regulated impaired autophagy and cellular senescence. The results showed that inhibited methylation with Adox or targeted SUV39H2 inhibition could delay IDD progression. Mechanistically, the K141 methylation of PPP1CA mediated by SUV39H2 weakens the phosphatase activity of the PPP1CA/PPP1R9B complex that binds to TFEB, which results in decreased TFEB activity (Fig. 9).

IDD is a complex process that is strongly associated with aging-related diseases, because NPC dysfunction due to senescence is a fundamental pathogenic mechanism of both disease states [27, 28]. Senescent resident cell populations and abnormally elevated inflammatory cytokine production combine to form a chronic inflammatory microenvironment that accelerates the IDD process [29–31]. As an important player in the maintenance of cellular and organismal homeostasis, autophagy is an intracellular molecular pathway that dynamically responds to environmental challenges [7]. Mounting evidence suggests that impaired autophagy is also strongly associated with the pathogenesis of aging-related diseases and that decreased autophagy leads to senescence and accelerates the IDD process [32, 33]. In our study, we revealed that impaired autophagy and NPC senescence may be caused by reduced TFEB activity during IDD progression. Furthermore, we identified SUV39H2 as a regulator of senescence, and its mediated methylation events impact TFEB dephosphorylation and subsequent activation. Genetic or pharmacologic targeting of methylation restored TFEB activity, alleviated impaired autophagy and senescence in NPCs and mitigated the IDD process. Our findings may provide new potential therapeutic targets for IDD intervention.

TFEB activity is regulated by multiple PTMs, and phosphorylation plays an important role in TFEB activity [13]. Phosphorylated TFEB is inactivated and retained in the cytoplasm. Conversely, dephosphorylation allows TFEB to enter the nucleus and activate its downstream target genes [34]. Either hyperactive phosphorylation or blocked dephosphorylation can lead to decreased TFEB activity and subsequent impaired autophagy. Previous studies have shown that mTORC1, a major kinase complex responsible for TFEB phosphorylation, is aberrantly activated in IDD tissues [35]. Thus, decreased TFEB activity in NPCs may be partially attributed to aberrant mTORC1 activation. We also identified PPP1CA/PPP1R9B as a novel phosphatase complex that promotes dephosphorylation-dependent TFEB nuclear translocation and downstream signaling. The association of the PPP1CA/PPP1R9B complex with TFEB was weakened in degenerated NPCs, resulting in the suppression of TFEB. These observations indicate that PPP1CA/PPP1R9B-regulated dephosphorylation is an alternative mechanism underlying TFEB activation and that the blocked dephosphorylation process of TFEB contributes greatly to its reduced activity in IDD.

Methylation is a universal PTM of proteins and is catalyzed by specific methyltransferases. SUV39H2 is a methyltransferase responsible for the methylation of histone H3 in lysine 9 and plays a vital role in the maintenance of heterochromatin and gene repression [36]. Recently, non-histone methylation has been widely identified as a regulator of various cellular signal transduction pathways. Indeed, SUV39H2 is also involved in the methylation of non-histone proteins such as LSD1 [37]. Notably, we found an interaction between SUV39H2 and PPP1CA in NPCs, and SUV39H2 could methylate PPP1CA at K141. PPP1CA is a major protein phosphatase that is commonly expressed in most cell types. The PPP1CA holoenzyme consists of a catalytic subunit and variable binding partners, known as regulatory subunits, which are capable of binding the catalytic subunit to a specific substrate. In our study, we observed that SUV39H2-mediated PPP1CA K141 methylation disrupts the interaction between PPP1CA and PPP1R9B and hinders the recruitment of PPP1CA to TFEB. Thus, we found crosstalk between methylation and phosphorylation that regulates TFEB activation from the perspective of protein‒protein interactions.

In summary, we identified SUV39H2-mediated PPP1CA K141 methylation as an initiating event of NPC senescence. NPC senescence depends on PPP1CA K141 methylation disrupting the interaction of PPP1CA/PPP1R9B and TFEB, ultimately leading to reduced TFEB activity and impaired autophagy. Importantly, we reveal that inhibiting the methylation process or targeting SUV39H2 could mitigate the progression of IDD and provide a potential therapeutic strategy for IDD intervention.

## Methods

### Human NP tissues

Human NP tissues were obtained from 14 patients who suffered from thoracolumbar fracture, scoliosis or IDD and underwent spinal surgery. Information on these patients is listed in Table S1. The tissues were used to perform immunoblot analysis or histological analysis. All the studies involving human tissues were approved by the Ethics Committee of Tongji Medical College, Huazhong University of Science and Technology (No. S341).

### Animal model and intradiscal injection

Three-month-old male Sprague‒Dawley rats (SD rats, 200 ± 20 g) were purchased from the Animal Experimentation Centre of Huazhong University of Science and Technology. The number of animals per group (*n*  =  5) in each experiment were determined following the previous studied. The animals were housed in an SPF class animal house and given adequate water, feed and light. All animal experiments were approved by the committee of Tongji Medical College, Huazhong University of Science and Technology (No. S2394), and all procedures were performed in accordance with the Declaration of Helsinki. No randomization method was used.

After weighing all experimental rats, the rats were anesthetized by intraperitoneal injection of 3% pentobarbital. After successful anesthesia, percutaneous puncture was performed using a 20-gauge needle at localized Co6-7, Co7-8 and Co8-9. The needle was punctured vertically through the tail of the rat and through the contralateral skin, rotated 360° and held for 30 s. The needle was then withdrawn. The puncture needle was inserted through the center of the disc and perpendicular to the longitudinal axis of the disc. The depth of penetration was controlled at 5 mm so that the tip of the needle reached the center of the NP region. The experimental animals were given adequate chow and sterile water after the operation, and the rats were closely monitored for one week after the operation for postoperative urinary retention, infection and other complications.

For Adox intervention, SD rats were randomly divided into 3 groups of 5 rats each. Two microliters of solution was injected into the center of the NP region by using a 31-gauge needle, keeping the needle in the disc for 10 s, and repeated injections were administered every week for 4 weeks.

For the intradiscal injection of AAV vectors, SD rats were randomly divided into 3 groups of 5 rats each. Two kinds of solutions were prepared for intradiscal injection: AAV Scrambled and AAV shSUV39H2. Two microliters of the indicated solution was slowly injected into the center of the NP region through a 31-gauge needle, keeping the needle in the disc for 10 s, and injections were repeated every week for 4 weeks.

We injected the indicated drugs into the discs of the rats individually by group and kept them in standard housing for one month. At 4 weeks postoperatively, the rats were sacrificed for radiographic and histological analyses. Treatment assignment is performed in a blinded manner.

### RT‒qPCR

Total RNA was extracted from NPCs by using TRIzol reagent (Thermo Fisher, USA). cDNA was synthesized using a cDNA Synthesis Kit (Vazyme, China). RT‒qPCR was performed using RT SuperMix for qPCR (Vazyme, China). Information on the primers used for RT‒qPCR is listed in Supplementary Table 2.

### Immunoprecipitation and Western blot analyses

After the cells were treated as indicated, they were washed twice with precooled PBS, lysed on ice by adding NP-40 for 30 min and centrifuged at 12,000 rpm for 25 min at 4 °C. The supernatant was transferred to a new 1.5 ml EP tube, 10% was aspirated for use as input, and the remaining 90% of the supernatant was added to the primary antibody and Protein A/G beads and incubated at 4 °C. Next, 1x SDS loading buffer was added to the resulting immunoprecipitate and denatured at 97 °C for 5 min. Western blot analysis was performed as previously described.

### LC‒MS/MS

Co-IPs of endogenous human protein of NP cells were conducted using anti-TFEB/ PPP1CA/ SUV39H2 antibody with protein A/G beads (MedChemExpres, USA) at 4 °C overnight on a rotator. After washed 5 times with pre-cooled NP-40 lysis buffer, immunoprecipitates were eluted with 1×loading buffer (Boster, USA) and separated SDS-PAGE gel. According to the manufacture’s protocol, SDS-PAGE gel piece was decolorized, alkylated and enzymatically disintegrated. The samples were analyzed by liquid chromatography–tandem mass spectrometry (LC-MS/MS) on a Q Exactive mass spectrometer (Thermo Fisher Scientific). The peptides were extracted, identified using Orbitrap Fusion Lumos (Thermo Scientific, USA) and analyzed using Proteome Discovery 1.4, Mascot server (v2.3, Matrix Science, UK). The existence of monomethylation was verified on the basis of the occurrence of consecutive y or b ions and the mass increment of 14.016 Da on lysine residues. Data are available via ProteomeXchange with identifier PXD042438, PXD042152, PXD042397.

### Cellular fractionation for Western blot analysis

Protein extraction was conducted using a protein subcellular isolation kit (Solarbio, China). Western blot analysis was performed as previously described. GAPDH was used as the internal control for cytoplasmic proteins, while histone 3 was used as the internal control for nuclear proteins.

### Immunofluorescence

Cells were cultured on sterile crawl plates and subjected to the indicated treatments. Immunofluorescence assays were performed after the indicated treatment time. The medium was aspirated from the wells and washed 3 times with PBS solution for 3 min each time; 4% paraformaldehyde was used to fix the cells at room temperature for 15 min. Then, 0.5% Triton X-100 (PBS preparation) was used to permeabilize the cells at room temperature for 20 min, and immunofluorescence blocking solution was used to block the cells at room temperature for 30 minutes, followed by incubation with anti-TFEB (1:100, 4240, CST), PP1 (1:200, 67070-1-Ig, Proteintech), or PPP1R9B (1:100, 55129-1-AP, Proteintech), LAMP2 (1:100, ab25631, Abcam), TFE3 (1:100, 14480-1-AP, Proteintech), MITF (1:100, 13092-1-AP, Proteintech), Cathepsin B (1:200, 12216-1-AP, Proteintech) overnight at 4 °C. After incubation, the cells were washed three times with PBST solution for 3 min each time, and the fluorescent secondary antibody was added and incubated for 1 hour before nuclei were restained with DAPI (Invitrogen, USA). Fluorescent images were observed under a fluorescence microscope and photographed (Olympus, BX53).

### Tandem stubRFP-sensGFP-LC3 fluorescence microscopy

The stubRFP-sensGFP-LC3 Lentivirus (GeneChem, China) was used to assess autophagic flux. NPCs were spread onto 24-well plates at a density of 1 × 10^5^ cells/well 24 hours prior to lentivirus transfection. The density of NPCs at the time of lentiviral transfection was set at approximately 2 × 10^5^ cells/well. The next day, the medium was replaced with 2 mL of fresh medium containing 7 µg/mL polybrene, and 2 µl of virus suspension was added. After 24 hours, the medium containing the virus was replaced with fresh medium, and incubation was continued. The cells were observed daily under a fluorescence microscope. After the indicated treatment, the cells were obtained under an inverted fluorescence microscope.

### Statistical analysis

Each group was analyzed in no less than three separate experiments, and statistical analysis was performed using GraphPad Prism 9.0. Data are expressed as the mean ± standard deviation. Student’s *t-*test was conducted to compare data between two groups for between-group analysis. Data from more than two groups were analyzed using one-way analysis of variance (ANOVA) with a least significant difference test to determine whether there was a statistically significant difference between the control and experimental groups. *p* < 0.05 indicates a statistically significant difference (^*^P  <  0.05, ^**^P  <  0.01,^***^P  <  0.001, and^****^P  <  0.0001).

Additional methods and materials are included within the supplemental information.

## Supplementary information

SUPPLEMENTAL MATERIAL

Original western blot data

checklist

## Data Availability

Data are available upon reasonable request.
