# Supplementary material for: Lysine methylation of PPP1CA by the methyltransferase SUV39H2 disrupts TFEB-dependent autophagy and promotes intervertebral disc degeneration
Source: Cell Death Differ. 2023 Aug 21;30(9):2135–50. doi: 10.1038/s41418-023-01210-4 (PMC10482945; doi:10.1038/s41418-023-01210-4)
Supplement: Supplementary file 1 — SUPPLEMENTAL MATERIAL [file 41418_2023_1210_MOESM1_ESM.docx]

Supplementary Materials for

**Lysine methylation of PPP1CA by the methyltransferase SUV39H2 disrupts TEFB-dependent autophagy and promotes intervertebral disc degeneration**

Huaizhen Liang^1^, Rongjin Luo^1^, Gaocai Li^1^, Weifeng Zhang^1^, Dingchao Zhu^1^, Di Wu^1^, Xingyu Zhou^1^, Bide Tong^1^, Bingjin Wang^1^, Xiaobo Feng^1^, Kun Wang^1^, Yu Song^1*^, Cao Yang^1*^.

Correspondence to: caoyangunion@hust.edu.cn

**This PDF file includes:**

Materials and Methods

Figures. S1 to S8

Tables S1 to S3

Materials and Methods

**Cell culture and treatment**

The NP tissue samples were separated, cut into pieces, and treated with 0.2% collagenase type II (Invitrogen, Carlsbad, CA, USA) for 8 h at 37 ℃. The digest was centrifuged at 1200 rpm and then cultured in Dulbecco’s Modified Eagle’s Medium (DMEM; Gibco, Grand Island, NY, USA) with 10% fetal bovine serum (FBS; Invitrogen), 1% penicillin–streptomycin (Sigma), 2 mM glutamine (Sigma), and 50 μg/mL L-ascorbic acid (Sigma) at 37℃ in 5% CO2. When grew to confluence, the cells were digested by 0.25% trypsin/1 mM EDTA and passed for expansion. The NPCs from passage 2 were plated into experimental plates for following experiments.

HEK293T cell line was purchased from the American Type Tissue Culture Collection (ATTC) and cultured in DMEM (Gibco, Grand Island, NY, USA) supplemented with 10% fetal bovine serum (FBS; Invitrogen).

In some experiments, cells were stimulated by 50μM of TBHP (Sigma) in the culture medium for 24 h. For the purpose of inhibiting methylation, NPCs were treated with medium containing 100 μM AdOx (Sigma). For the purpose of inhibiting SUV39H2, NPCs or HEK293T cells were treated with medium containing 1 μM or 2 μM OTS186935 (MedChemExpress). For autophagy flux blockage, NPCs were treated with 100nM Bafilomycin A1 (MedChemExpress) or 20mM 3-MA (MedChemExpress). For mTORC1 inhibition, NPCs were treated with 500 Nm Torin1 (MedChemExpress).

**RNA Interference and Plasmid Transfection**

Knockdown of TFEB, PPP1CA, PPP1R9B and SUV39H2 in NPCs was realized by transfection with siRNA. After verified high silencing efficiency, the NP cells were then used in following treatment. Vectors of lentivirus pLKO.1 were used in which shRNA against PPP1CA was cloned. The adeno-associated virus (AAVs) containing short hairpin RNA of SUV39H2 or AAV-Scrambled) were generated using the pAAV2/5-CMV system (OBIO, China) according to the manufacture’s protocols. Further information of siRNA and shRNA sequences is provided in Table S2.

To generate the mammalian expression plasmids, human wild-type TFEB cDNA (NM_001271944.2) and mutant (S211A) was cloned into the pHBLV-CMV (Flag-tag); human wild-type PPP1CA cDNA (NM_002708) and mutants (K141R, K141M) were cloned into the GV658-CMV (His-tag); human PPP1R9B cDNA (NM_032595.5) was cloned into the pCDNA3.1-CMV-ZsGreen (GST-tag); human SUV39H2 cDNA (NM_032595.5) was cloned into the pOTB7-CMV-mCheery (MBP-tag). To efficiently transient transfection, NP cells and HEK293T cells were transfected with the previous plasmids using Lipofectamine™ 2000 (Invitrogen, USA) for 72 h.

**Western blot**

Samples were lysed by RIPA (Beyotime, P0013B, Shanghai, China) and their protein contents were measured using the Micro BCA Protein Assay Kit (Beyotime, P0010S, Shanghai, China). SDS-polyacrylamide gel electrophoresis gels were used for electrophoresis and then transferred to PVDF membranes. The membranes were incubated with the specific primary antibody (The primary antibodies used were listed in Table S3) overnight at 4 ℃ after blocking with Blocking buffer and HRP-conjugated Affinipure Goat Anti-Rabbit IgG (SA00001-2, Proteintech,1:10000) and HRP-conjugated Affinipure Goat Anti-Mouse IgG (SA00001-1, Proteintech, 1:1000) were added. Protein expression was visualized using enhanced chemiluminescence reagents (Affinity, KF001, Nanjing, China) and the ChemiDoc MP Imaging System (Bio-Rad, 12003154 Hercules, CA, USA). And Image J (V1.52v) was used for semi-quantification of the expression of proteins.

**EdU Incorperation Assay**

EdU labeling was performed to examine the proliferation status of NPCs. NPCs were exposed to 25×10^6^ M of 5-ethynyl-2′-deoxyuridine (EdU, RiboBio, C10338, Guangzhou, China) for 12h at 37°C and fixed in 4% paraformaldehyde. NPCs were then permeabilized using 0.5% Triton-X-100 and then reacted with Apollo488 for 30 mins. subsequently, Hoechst 33342 was used to stain the DNA contents of the cells for 30 min, and images were visualized and captured using a microscope (Olympus, BX53). EdU positive cells were analyzed using Image J. The experiments were replicated three times.

**ELISA assay**

Briefly, the relative secretion levels of inflammatory cytokines (IL-1β, IFN-β1, IFN-γ, TGF-β1, IL-6, TNF-α) were detected in NP cell culture supernatants by ELISA using commercial kits (Elabscience) according to the protocols of the manufacturer.

**SA-β-galactosidase Staining**

NPCs were first fixed with special fixative for 20 min, at room temperature for 15 mins after corresponding disposal and then stained using fresh staining solution at 37°C for 12 hours. Images were captured under a microscope (Olympus, BX53; Melville, NY, USA).

**Lysosomal staining**

LysoTracker (LysoTracker Red, Yeasen, China) were employed to label lysosome in NPCs. NPCs were cultured in poly-d-lysine coated glass-bottom dish (35 mm). NPCs were washed and incubated with 50 nM LysoTracker Red DND-99 in HBSS for 30 min at 37°C. The cells were then rinsed twice with warm HBSS, and the live cells were immediately examined using a microscope (Olympus, BX53).

**Magic Red assay**

For the Magic Red Cathepsin B assay, the NPCs were loaded with Magic Red Cathepsin B substrate (#937, Immunochemistry Technologies) for ~40 min according to the manufacturer’s instructions. Images were acquired using microscopy (Olympus, BX53).

**Immunohistochemistry (IHC)**

For IHC, paraffin-embedded tissue sections were deparaffinized with xylene and rehydrated with an alcohol gradient and water. Sections were incubated with primary antibodies p16 (diluted 1:100, Affinity, catalogue number: #80772), p21 (diluted 1:300, Proteintech, catalogue number: 114 #2947), LC3 (diluted 1:300, Proteintech, catalogue number: ab39012), p62 (diluted 1:200, Proteintech, catalogue number: ab39012), SUV39H2 (diluted 1:100, Proteintech, catalogue number: ab39012) at room temperature for 1h and biotin-labelled secondary antibodies for 30min, and then stained with Vectastain ABC kit and DAB peroxidase substrate kit (Boster, AR1000).

**Histological and Radiographic Evaluation and Analysis**

One months later, rats were sacrificed and histological and radiographic evaluation were performed. After X-ray and MRI examination, tails were fixed in 10% neutral-buffered formalin for 1 week and Midsagittal sections were stained with hematoxylin and Safranin O-fast green to evaluate the degeneration level. The histological evaluation was performed according to histologic grading system developed by Ji et al^1^. Radiographs were taken at 4 weeks after the puncture. The change in IVD height was evaluated by the disc height index (DHI) The change in IVD height was evaluated by the disc height index (DHI) Measurements of internal control discs were carried out together with their corresponding punctured discs. Disc height and the adjacent vertebral body heights were measured on the midline and 25% of the disc’s width from the midline on either side. The DHI was expressed as the mean of the 3 measurements from midline to the boundary of the central 50% of disc width divided by the mean of the 2 adjacent vertebral body heights. Changes in the DHI of punctured discs were expressed as a percentage (%DHI=post-punctured DHI/pre-punctured DHI ×100). MRI-based Pfirrmann grading system was applied to assess the degenerative grade of the NP specimens^2^.

**References**

1 Ji, M. L. *et al.* Preclinical development of a microRNA-based therapy for intervertebral disc degeneration. *Nature communications* **9**, 5051, doi:10.1038/s41467-018-07360-1 (2018).

2 Pfirrmann, C. W., Metzdorf, A., Zanetti, M., Hodler, J. & Boos, N. Magnetic resonance classification of lumbar intervertebral disc degeneration. *Spine* **26**, 1873-1878, doi:10.1097/00007632-200109010-00011 (2001).

Figure S1


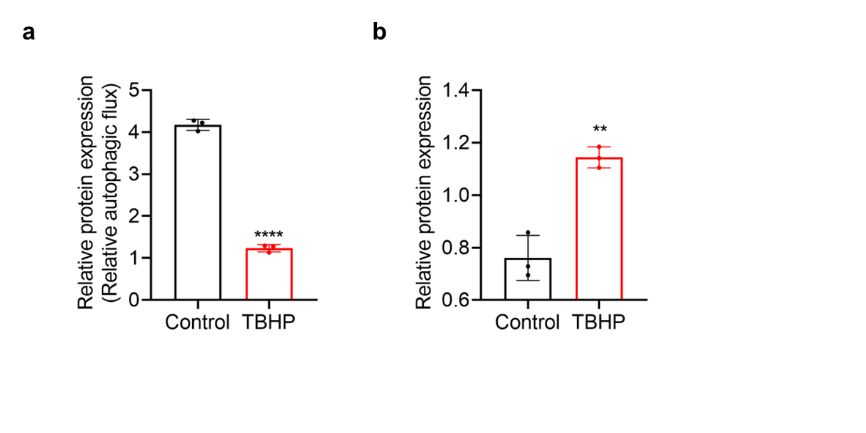


**Figure S1. (a)** Western blot analysis of LC3 in human NPCs with the indicated treatment. **(b)** Western blot analysis of p62 in human NPCs with the indicated treatment. Data are expressed as mean ± SD, n=3. **p < 0.01, ****p < 0.0001, ns not significant, two-tailed unpaired t test.

Figure S2.


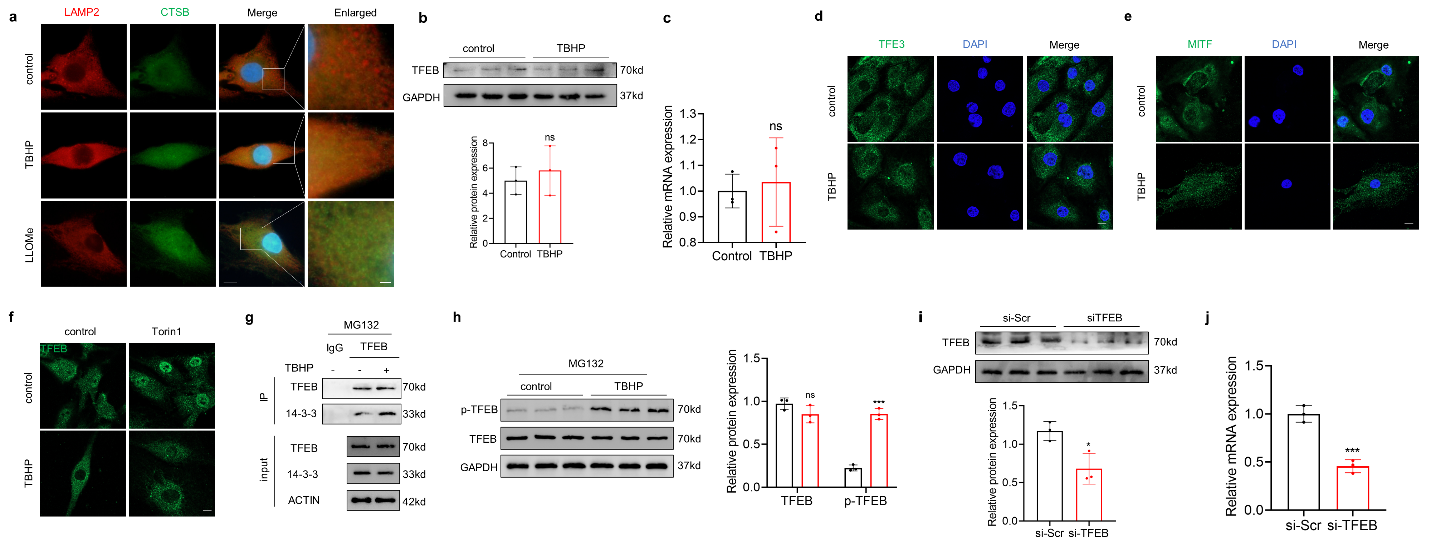


**Figure S2. (a)** IF analysis of the colocalization of LAMP2 with CTSB in NPCs treated with the indicated treatment (Scale bar: 10 μm, 2μm). **(b, c)** TFEB protein and mRNA level in human NPCs with the indicated treatment. **(d)** IF staining of TFE3 in NPCs with the indicated treatment (Scale bar: 10 μm). **(e)** IF staining of MITF in NPCs with the indicated treatment (Scale bar: 10 μm). **(f)** IF staining of TFEB in NPCs with the indicated treatment (Scale bar: 10 μm). (**g**) Co-IP analysis of the interaction of TFEB with 14-3-3 in NPCs with the indicated treatment. (**h**) Western blot analysis of p-TFEB and TFEB in human NPCs with the indicated treatment. **(i, j)** Knockdown efficiency of TFEB in human NPCs using specific siRNA confirmed by western blot and RT-qPCR. Data are expressed as mean ± SD. *p < 0.05, **p < 0.01, ***p < 0.001, ****p < 0.0001, ns not significant, two-tailed unpaired t test.

Figure S3.


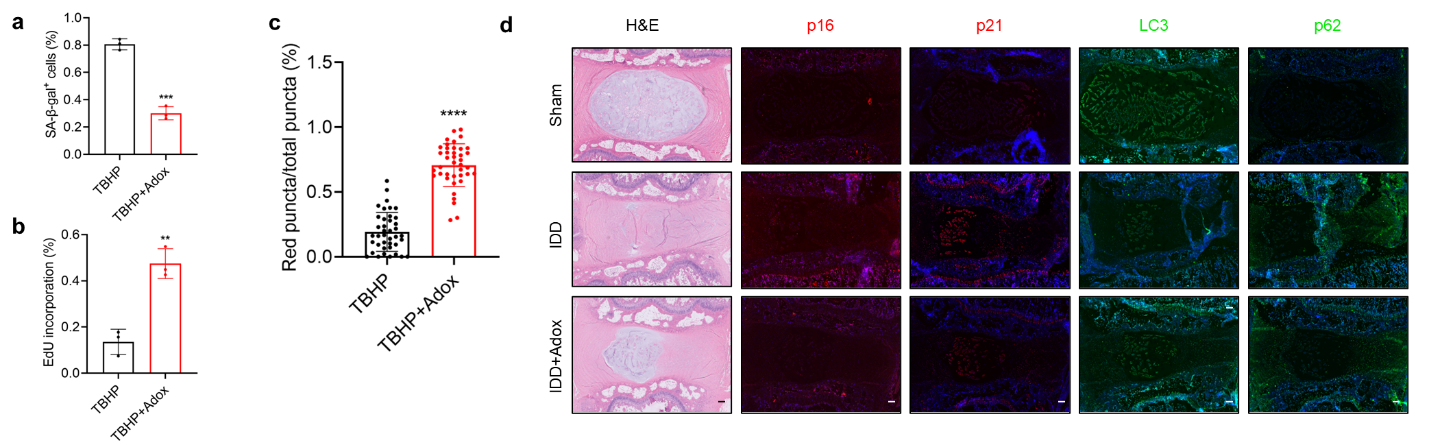


**Figure S3. (a)**SA‐β‐gal activity analysis of NPCs with the indicated treatment. **(b)** EdU incorporation analysis of NPCs with the indicated treatment. **(c)** Ratio of red: total puncta in NPCs treated with 100 μM AdOx for 24 h. **(d)** H&E staining IF staining of p16, p21, LC3, p62 of coccygeal vertebrae from rats with the indicated treatment (Scale bar: 200 μm). Data are expressed as mean ± SD. **p < 0.01, ***p < 0.001, ****p < 0.0001, two-tailed unpaired t test.

Figure S4.


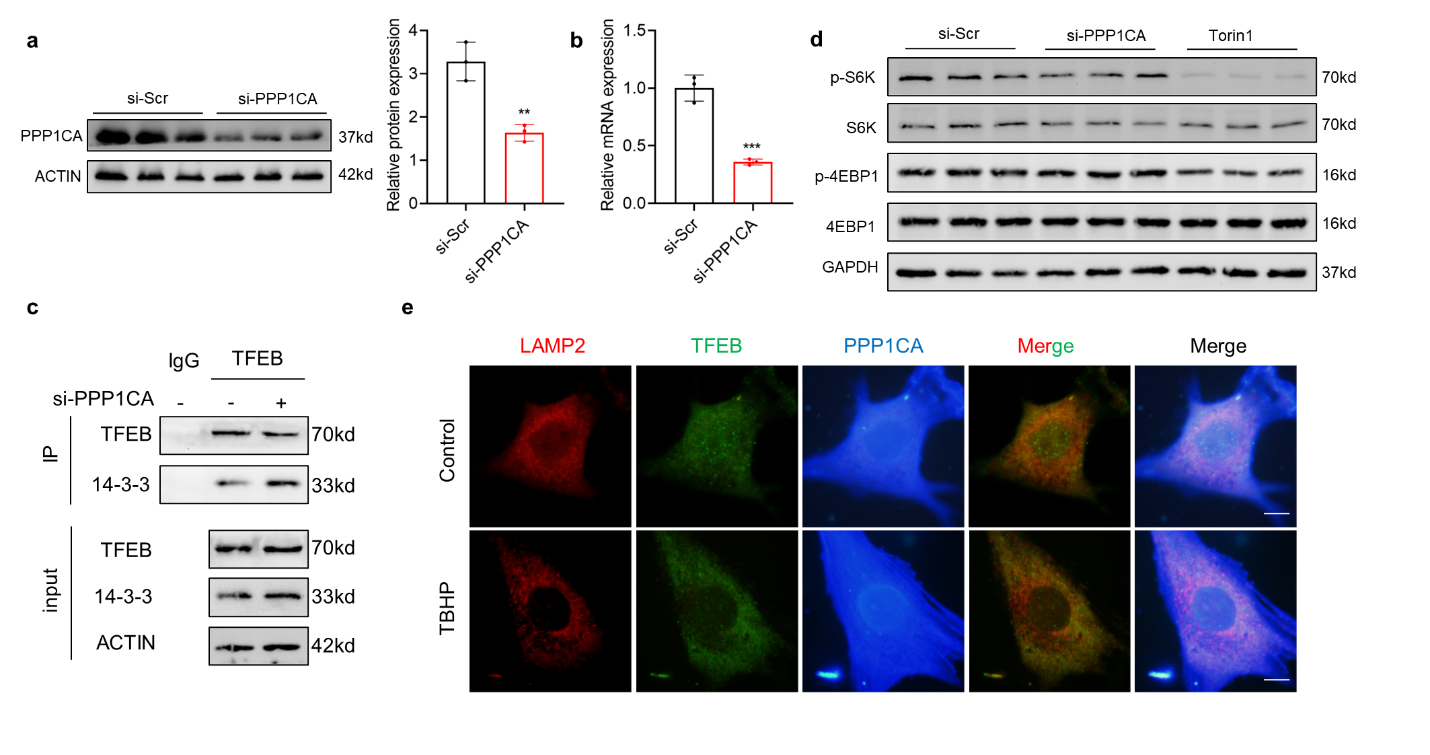


**Figure S4. (a, b)** Knockdown efficiency of PPP1CA in human NPCs using specific siRNA confirmed by western blot and RT-qPCR. (**c**) Co-IP analysis of the interaction of TFEB with 14-3-3 in NPCs with the indicated treatment. (**d**) Western blot analysis of S6K, p- S6K, 4EBP1, p-4EBP1 in human NPCs with the indicated treatment. (**e**) IF analysis of the colocalization of LAMP2, TFEB, PPP1CA in NPCs treated with the indicated treatment (Scale bar: 10 μm). **p < 0.01, ***p < 0.001, two-tailed unpaired t test.

Figure S5.


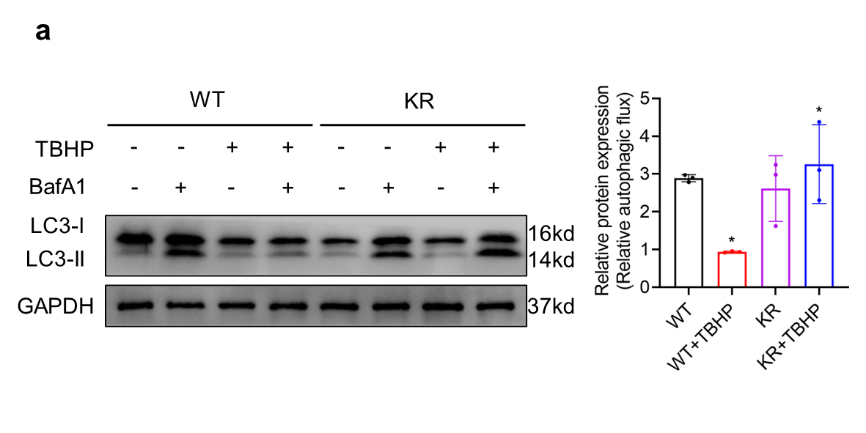


**Figure S5. (a)** Western blot analysis of LC3 in NPCs reconstituted expression of wild-type PPP1CA or KR mutant with the indicated treatment. *p < 0.05, one-way ANOVA.

Figure S6.


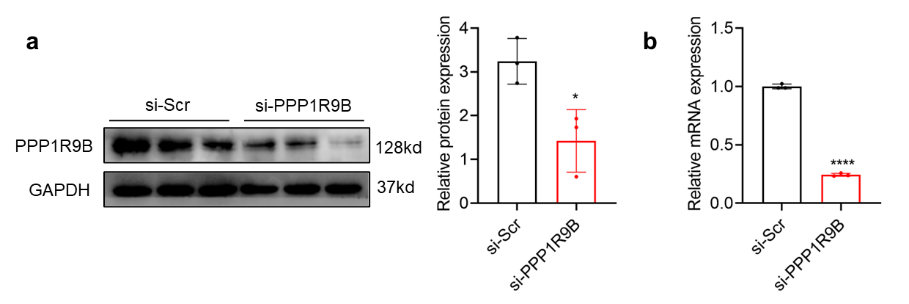


**Figure S6. (a, b)** Knockdown efficiency of PPP1R9B in human NPCs using specific siRNA confirmed by western blot and RT-qPCR. *p < 0.05, ****p < 0.0001, two-tailed unpaired t test.

Figure S7.


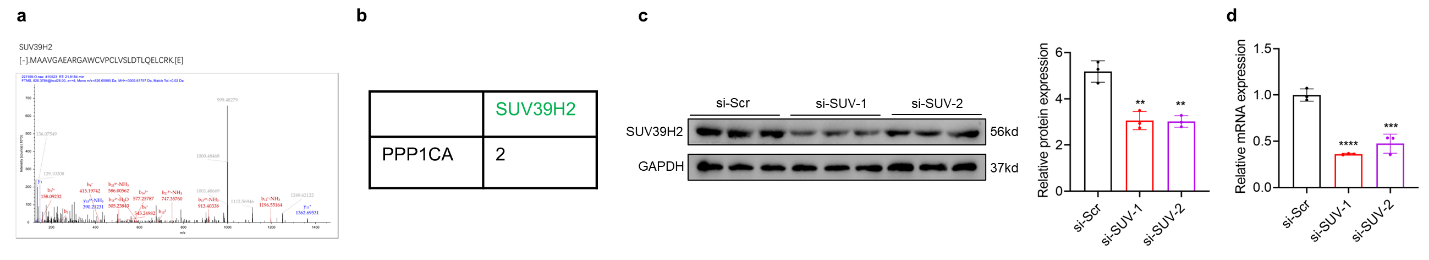


**Figure S7. (a)** LC-MS/MS spectrum of the tryptic peptide MAAVGAEARGAWCVPCLVSLDTLQELCRK. **(b)** Mass spectrometry identification of peptide counts by SUV39H2 immunoprecipitation **(c, d)** Knockdown efficiency of SUV39H2 in human NPCs using two specific siRNAs confirmed by western blot and RT-qPCR. **p < 0.01, **p < 0.001, ****p < 0.0001, and one-way ANOVA.

Figure S8.


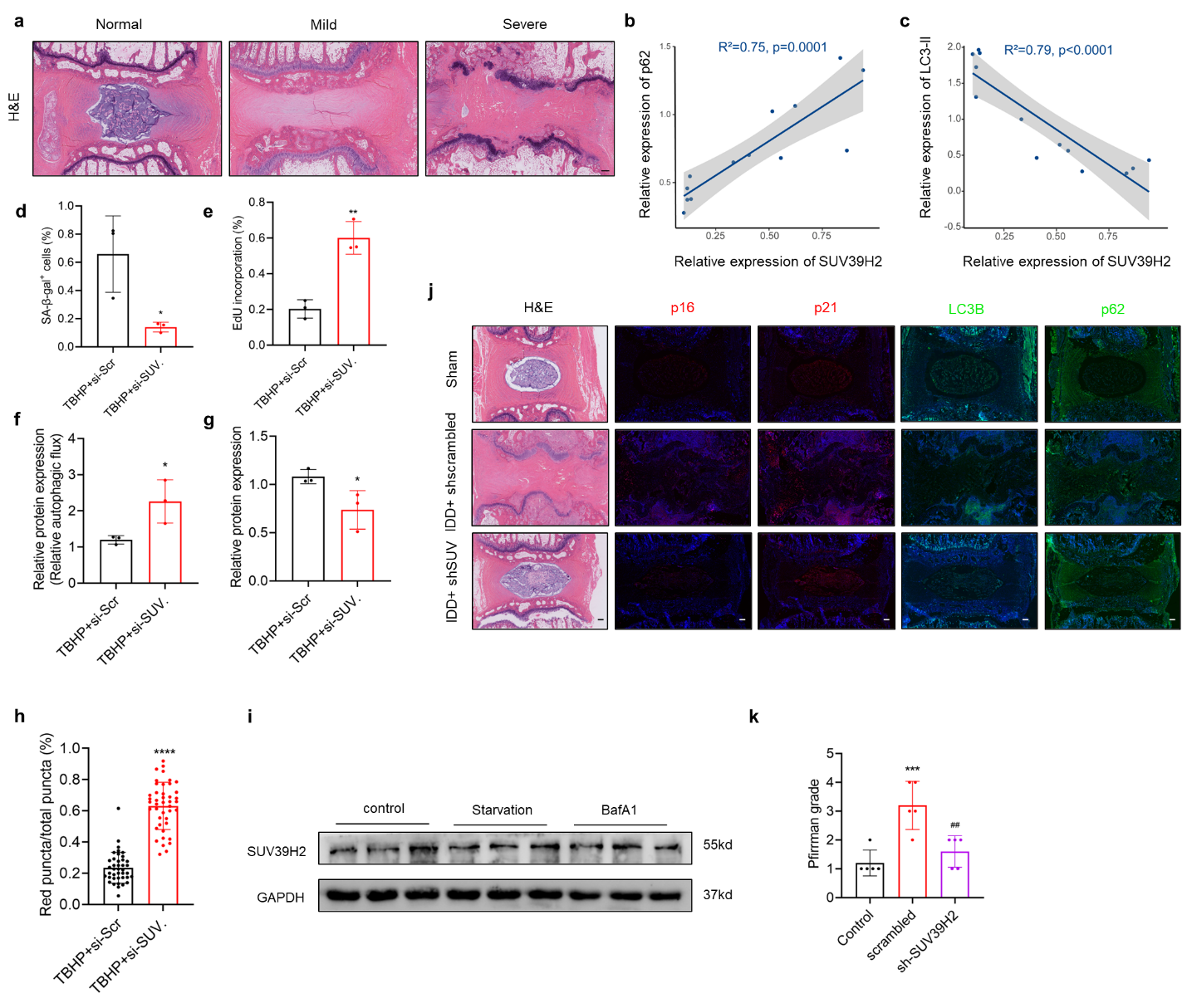


**Figure S8.** (**a**) H&E staining of coccygeal vertebrae from rats with normal, mild and severe degenerative degrees (Scale bar: 200 μm). (**b, c**) Linear regression analysis between the SUV39H2 protein levels and the LC3/p62 protein levels (**d**) SA‐β‐gal activity analysis of NPCs transfected with si-SUV39H2. **(e)** EdU incorporation analysis of NPCs transfected with si-SUV39H2. (**f**) Western blot analysis of LC3 in human NPCs with the indicated treatment. **(g)** Western blot analysis of p62 in human NPCs with the indicated treatment. (**h**) Ratio of red: total puncta in NPCs transfected with si-SUV39H2. (**i**) Western blot analysis of SUV39H2 in NPCs with indicated treatment. (**j**) H&E staining IF staining of p16, p21, LC3, p62 of coccygeal vertebrae from rats with the indicated treatment (Scale bar: 200 μm). (**k**) The Pfirrmann MRI grade scores of coccygeal vertebrae from rats with the indicated treatment. (n = 5). Data are expressed as mean ± SD. *p < 0.05, **p < 0.01, ***p < 0.001, ****p < 0.0001, two-tailed unpaired t test (**b-d**) and one-way ANOVA (**e**).

Table S1. Patient demographics

| **Case Number**  **number** | **Gender** | **Age (years)** | **Pfirrmann grade** | **Diagnosis** |
| --- | --- | --- | --- | --- |
| Case 1 | Female | 12 | I | Idiopathic scolios |
| Case 2 | male | 17 | I | Idiopathic scolios |
| Case 3 | Female | 11 | I | Idiopathic scolios |
| Case 4 | Female | 13 | I | Idiopathic scolios |
| Case 5 | Female | 14 | I | Idiopathic scolios |
| Case 6 | male | 26 | II | Idiopathic scolios |
| Case 7 | male | 15 | I | Idiopathic scolios |
| Case 8 | male | 40 | III | Lumbar disc herniation |
| Case 9 | Female | 49 | IV | Lumbar disc herniation |
| Case 10 | Female | 32 | III | Lumbar disc herniation |
| Case 11 | Female | 23 | III | Lumbar disc herniation |
| Case 12 | male | 27 | III | Lumbar disc herniation |
| Case 13 | Female | 64 | IV | Lumbar spinal stenosis |
| Case 14 | Female | 22 | II | Lumbar disc herniation |
| Case 15 | Female | 31 | II | Lumbar disc herniation |
| Case 16 | male | 32 | II | Lumbar disc herniation |
| Case 17 | male | 45 | III | Lumbar disc herniation |
| Case 18 | Female | 64 | IV | Lumbar spinal stenosis |
| Case 19 | male | 54 | IV | Lumbar spinal stenosis |

Table S2. Oligonucleotide sequences used in this study

| **Oligonucleotide**  **e name name** | **Sense (5’-3’)** | **Antisense (3’-5’)** |
| --- | --- | --- |
| **Primers for qPCR** | | |
| Homo PPP1R9B | GCCCAGCTAATTCAGCAGAC | GGAGCTCCTTGAACTTGTGC |
| Homo PP1 | GCCTGCTGGAAGGTGACATA | GCCTGCTGGAAGGTGACATA |
| Homo SUV39H2 | GCGCGAGGAGCTTGGT | TCTGGCCATCCTTTCCATTT |
| Homo TPP1 | GATCCCAGCTCTCCTCAATAC | GCCATTTTTGCACCGTGTG |
| Homo CTSF | ACAGAGGAGGAGTTCCGCACTA | GCTTGCTTCATCTTGTTGCCA |
| Homo MCOLN1 | GAGTGGGTGCGACAAGTTTC | TGTTCTCTTCCCGGAATGTC |
| Homo CTSD | CTTCGACAACCTGATGCAGC | TACTTGGAGTCTGTGCCACC |
| Homo LAMP1 | ACGTTACAGCGTCCAGCTCAT | TCTTTGGAGCTCGCATTGG |
| Homo WIPI1 | AGTCAGTCACACAAAACCACG | AGAGCACATAGACCTGTTGGG |
| Homo ATG9A | CTGCCCTTCCGTATTGCAC | CTCACGTTTGTGGATGCAGAT |
| Homo ATG10 | AGACCATCAAAGGACTGTTCTGA | GGGTAGATGCTCCTAGATGTGAC |
| Homo p62 | CAATGTTGGTTTCACTGAGAGC | ATACACTTGTTAGGAGGGACAG |
| Homo LC3B | TGTTGTTACGGAAAGCAGCAG | AACTGACATGCACTTGGTGTG |
| Homo ATG7 | TGGGAAGCCATAAAGTCAGG | GGCAGCAAAACCAGTAGTAGAA |
| Homo GAPDH | CAAGAAGGTGAAGCAGG | TCAAAGGTGGAGGAGTGGGT |
| **siRNA and shRNA sequence** | | |
| Homo-siControl | CAAGCCUUAGUGUUGGUUCACCUUU | AAAGGUGAACCAACACUAAGGCUUG |
| Homo-siTFEB | GAAAGGAGACGAAGGUUCA | UGAACCUUCGUCUCCUUUC |
| Homo-siPPP1CA | CCGCCAAAGCCAAGAAAUA | UAUUUCUUGGCUUUGGCGG |
| Homo-shPPP1CA | CCGCCAAAGCCAAGAAAUA | UAUUUCUUGGCUUUGGCGG |
| Homo-siPPP1R9B | UGUACUUGCGCUCCAGGGC | GCCCUGGAGCGCAAGUACA |
| Homo-siSUV39H2-1 | GGUCAGAACAGUAUGUAAA | UUUACAUACUGUUCUGACC |
| Homo-siSUV39H2-2 | UGGAAAGGAUGGCCAGAUU | AAUCUGGCCAUCCUUUCCA |
| Homo-siATG7 | CAACAUCCCUGGUUACAAG | CUUGUAACCAGGGAUGUUG |
| Rat-shSUV39H2 | GCTCCTGGGATCACCTTAAAC | GTTTAAGGTGATCCCAGGAGC |

Table S3. Antibody information

| **Antibody** | **Company** | **Catalog#** | **Application/ Dilution** |
| --- | --- | --- | --- |
| anti-p16INK4a | Affinity | AF5484 | IHC (1:100); WB (1: 500) |
| anti-p21 | CST | 2947 | IHC (1:300); WB (1: 500) |
| anti-p53 | CST | 2527 | WB (1: 1000) |
| anti-LC3 | Proteintech | 14600-1-AP | IHC (1:300); WB (1: 1000) |
| anti-p62 | Proteintech | 18420-1-AP | IHC (1:200); WB (1: 5000) |
| anti-TFEB | CST | 4240 | WB (1: 1000); IP (4 µg/1000 µg) |
| anti-TFEB | Proteintech | 13372-1-AP | WB (1: 1000) |
| anti-TFEB | Santa Cruz | sc-166736 | IP (4 µg/1000 µg) |
| anti-Phospho-TFEB (Ser211) | Affinity | AF3708 | WB (1: 500) |
| anti-Histone-H3 | Proteintech | 17168-1-AP | WB (1: 2000) |
| anti-GAPDH | Proteintech | 60004-1-Ig | WB (1: 2000) |
| anti-β-actin | Proteintech | 66009-1-Ig | WB (1: 2000) |
| anti-PPP1CA | Proteintech | 67070-1-Ig | WB (1: 2000); IP (4 µg/1000 µg) |
| anti-Mono-Methyl Lysine | CST | 14679 | WB (1: 1000) |
| anti-PPP1R9B | Proteintech | 55129-1-AP | WB (1: 1000); IP (4 µg/1000 µg) |
| anti-SUV39H2 | Proteintech | 11338-1-AP | IHC (1:100); WB (1: 1000); IP (4 µg/1000 µg) |
| anti-G9a | Proteintech | 66689-1-Ig | WB (1: 2000) |
| anti-SETD1A | Proteintech | 67936-1-Ig | WB (1: 5000) |
| anti-Flag tag epitope | Proteintech | 66008-4-Ig | WB (1: 500); IP (4 µg/1000 µg) |
| anti-His tag | Proteintech | 66005-1-Ig | WB (1: 5000); IP (4 µg/1000 µg) |
| anti-GST tag | Proteintech | 66001-2-Ig | WB (1: 5000); IP (4 µg/1000 µg) |
| anti-MBP tag | Proteintech | 66003-1-Ig | WB (1: 1000); IP (4 µg/1000 µg) |
| anti-4EBP1 | Proteintech | 60246-1-Ig | WB (1: 1000) |
| anti-Phospho-S6K(Thr389) | Proteintech | 28735-1-AP | WB (1: 2000) |
| anti-Phospho-4EBP1 (Ser65/Thr70) | Affinity | AF2308 | WB (1: 1000) |
| anti-S6K | Proteintech | 14485-1-AP | WB (1: 2000) |
