# Supplementary material for: Lysine methylation of PPP1CA by the methyltransferase SUV39H2 disrupts TFEB-dependent autophagy and promotes intervertebral disc degeneration
Source: Cell Death Differ. 2023 Aug 21;30(9):2135–50. doi: 10.1038/s41418-023-01210-4 (PMC10482945; doi:10.1038/s41418-023-01210-4)
Supplement: Supplementary file 2 — Original western blot data [file 41418_2023_1210_MOESM2_ESM.docx]

Original western blot data

**Figure 1**

Panel c:

p53 p21


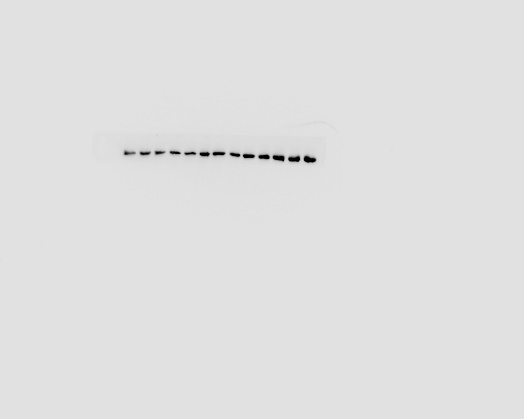

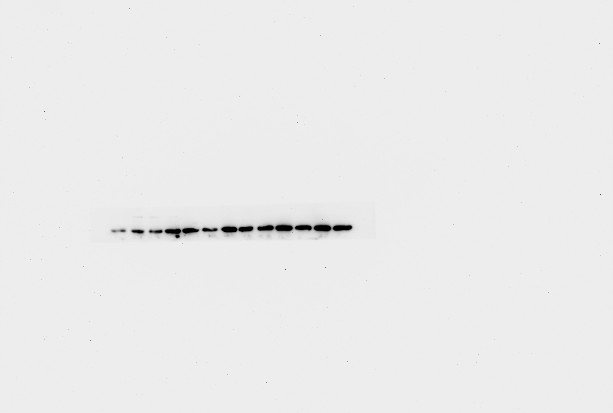


p16 TFEB


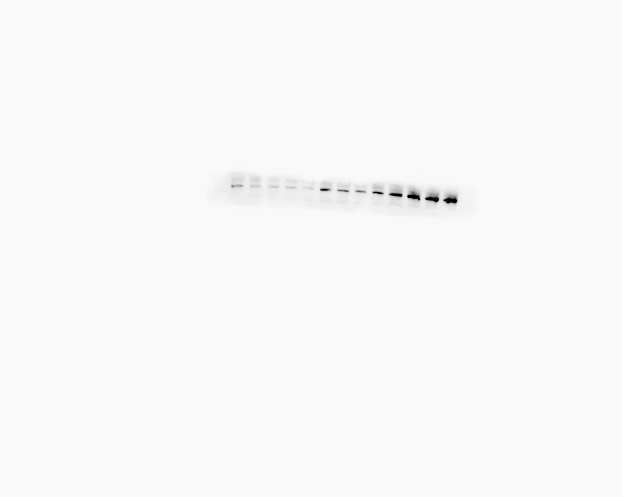

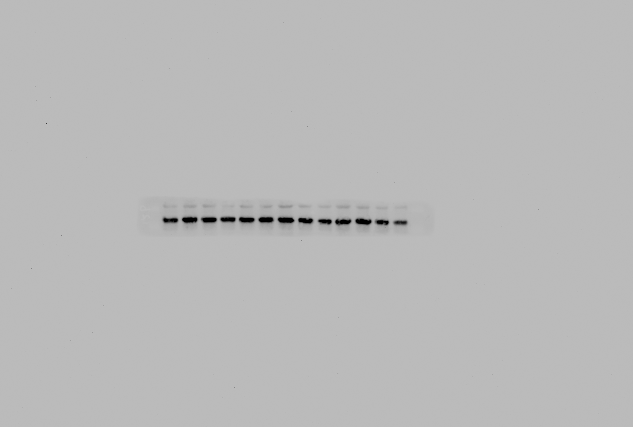


p-TFEB p62


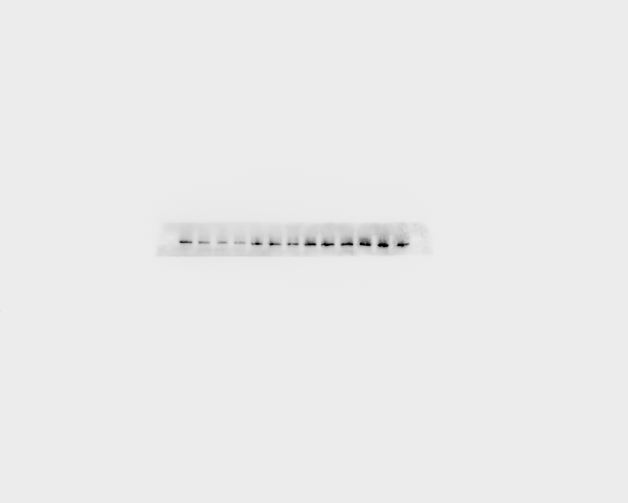

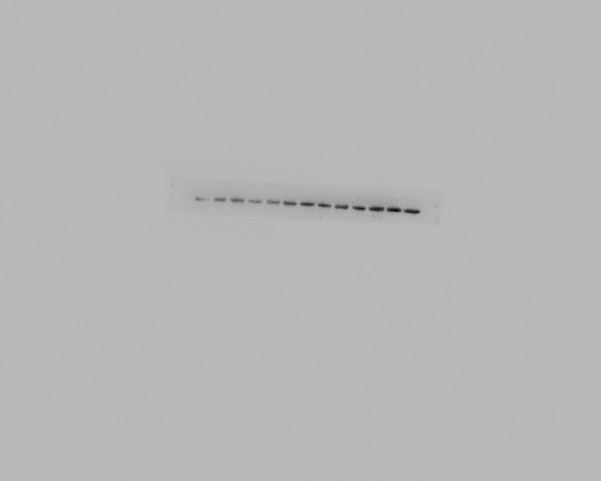


LC3 GAPDH


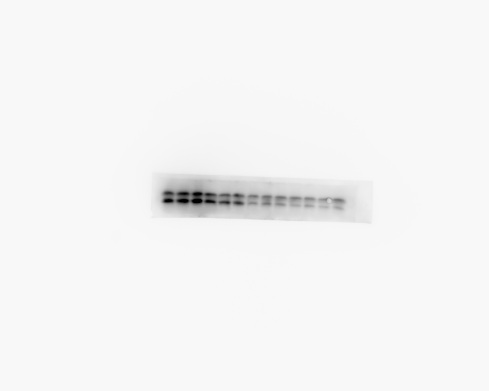

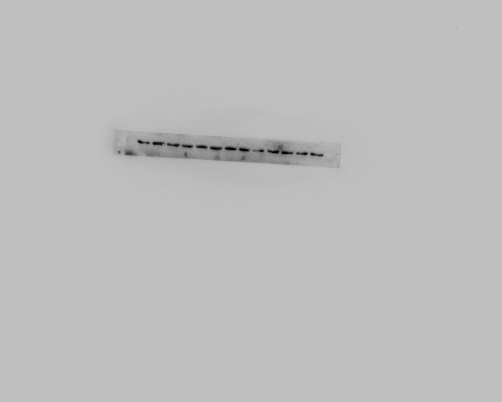


Panel d:

p53 p21


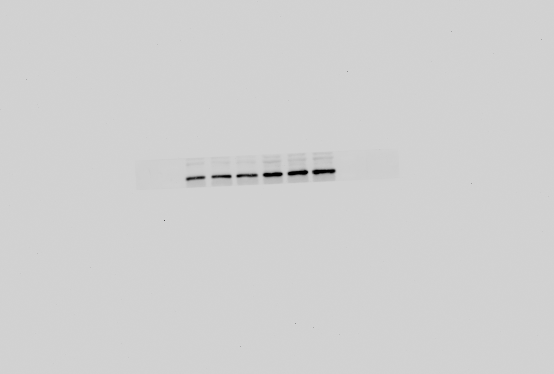

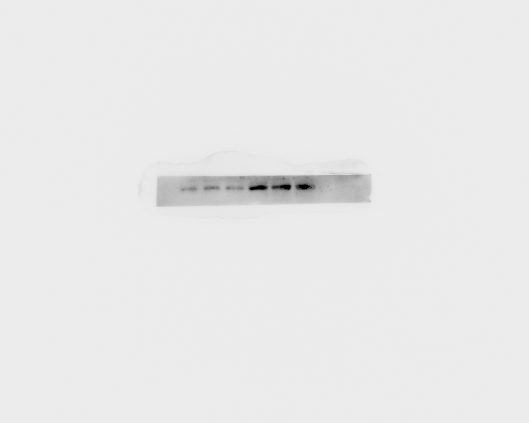


p16 GAPDH


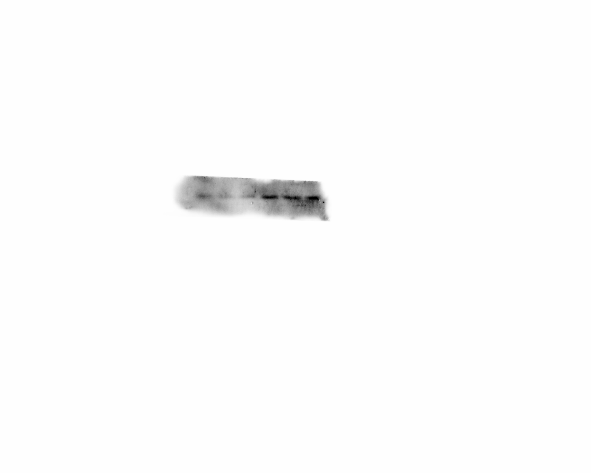

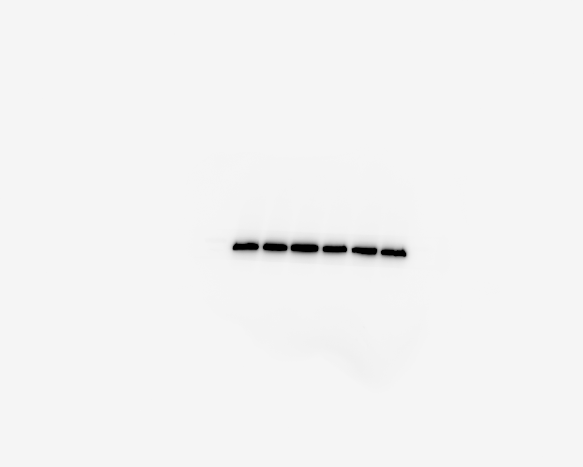


Panel j

LC3 GAPDH


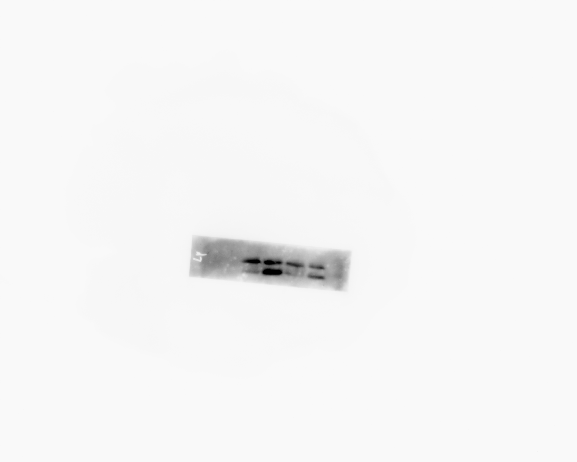

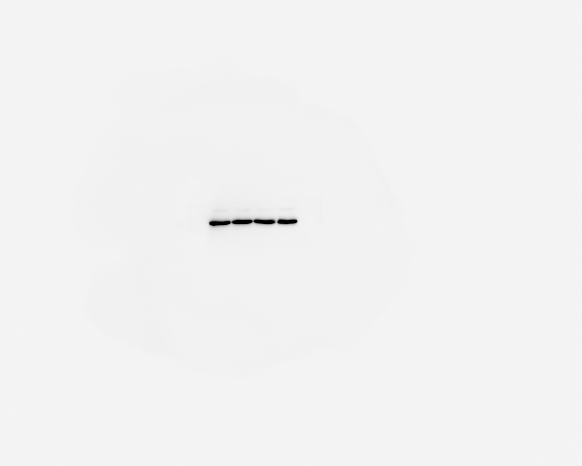


Panel k:

p62 GAPDH


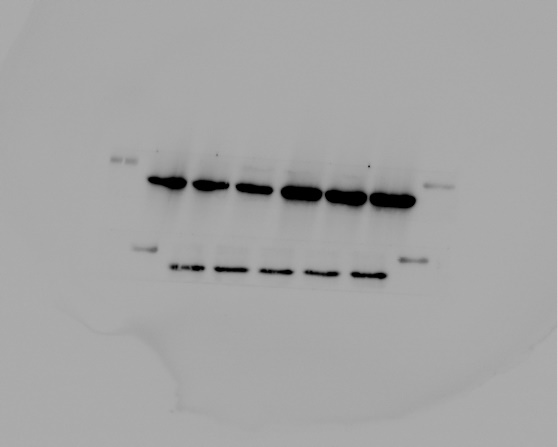

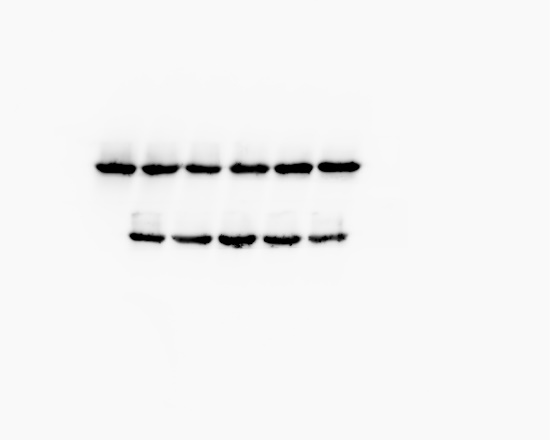


Panel m:

p53 p21


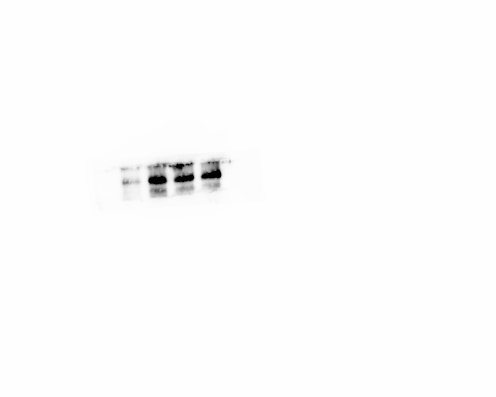

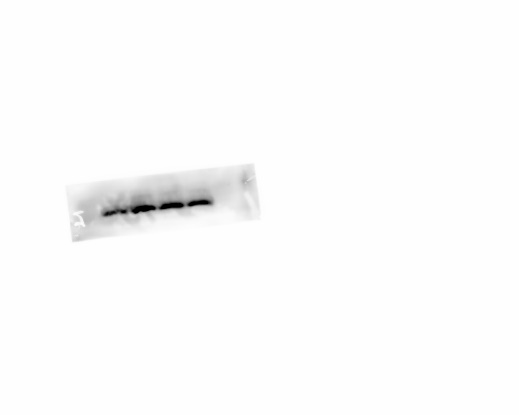


p16 GAPDH


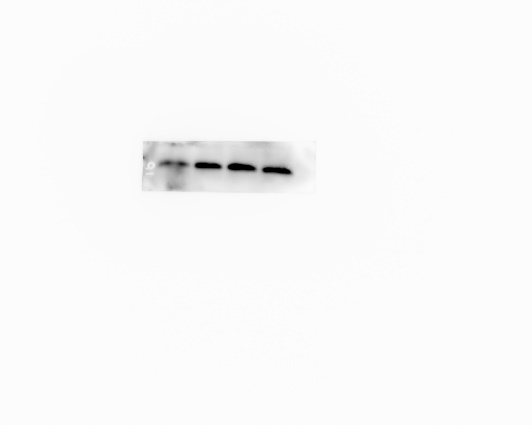

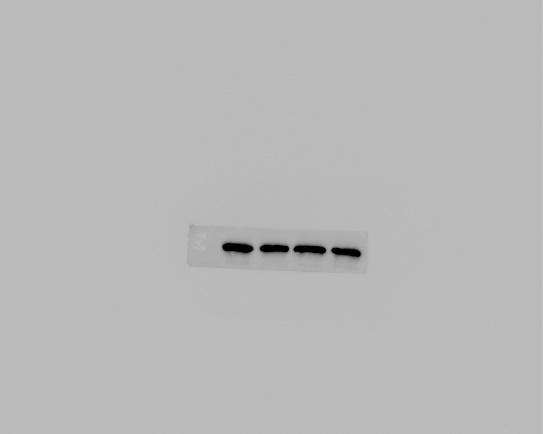


**Figure 2**

Panel d:

TFEB GAPDH


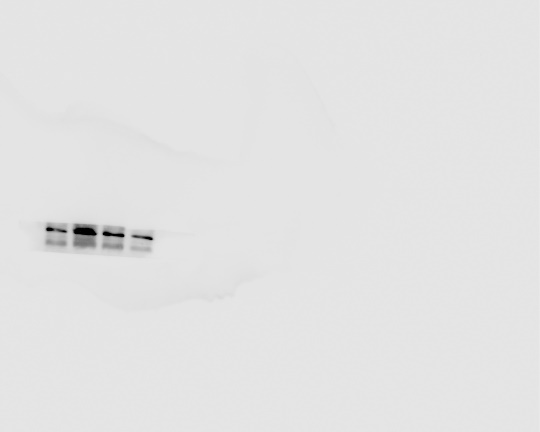

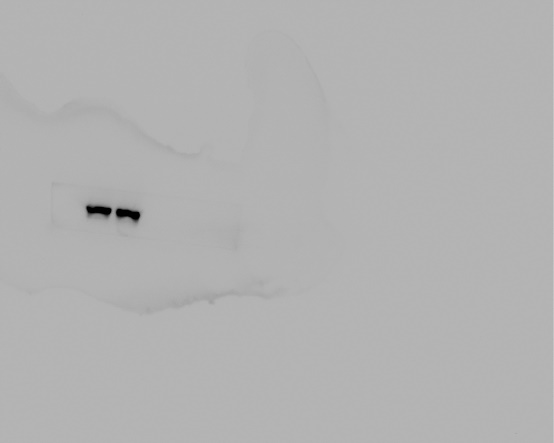


H3 p-TFEB


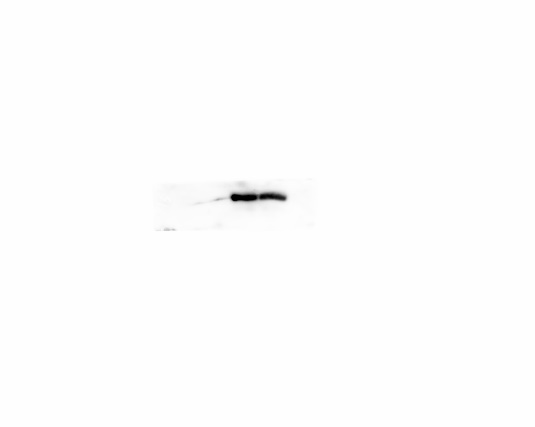

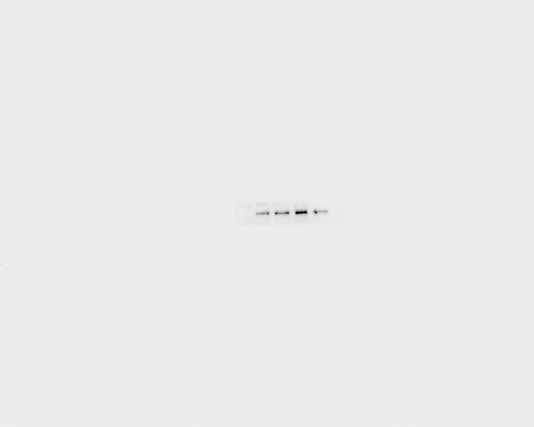


Panel e:

p-TFEB TFEB


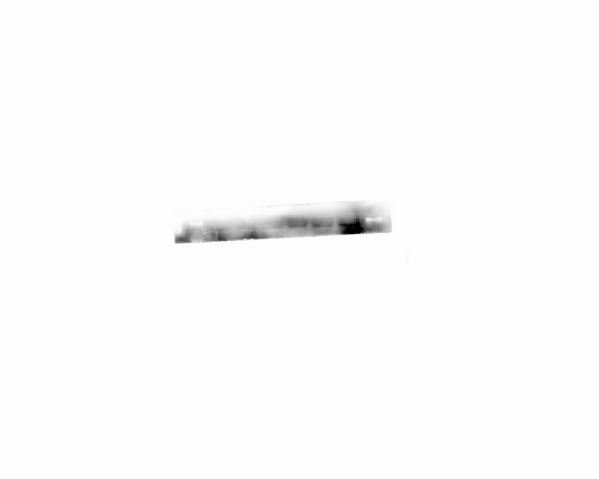

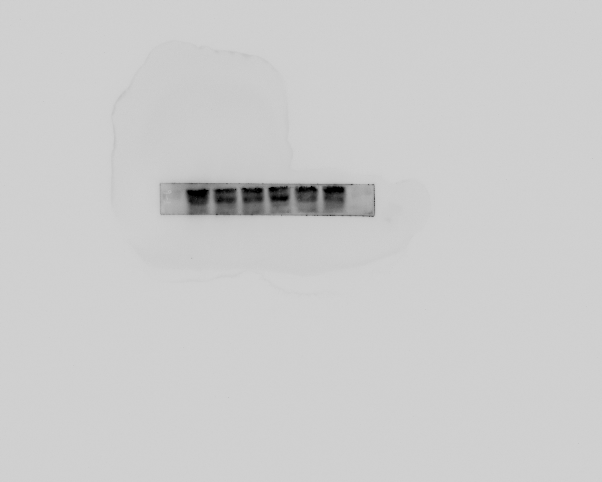


GAPDH


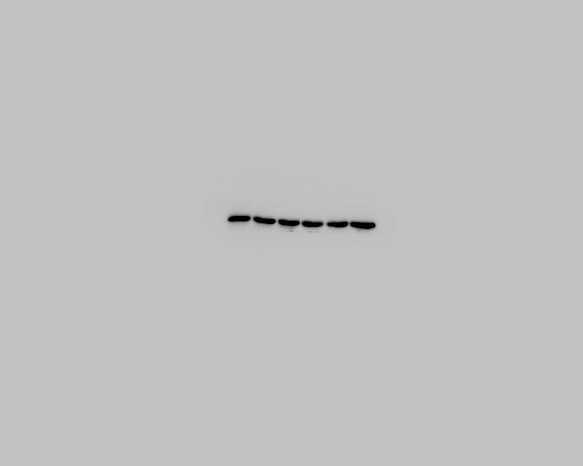


Panel f:

p53 p21


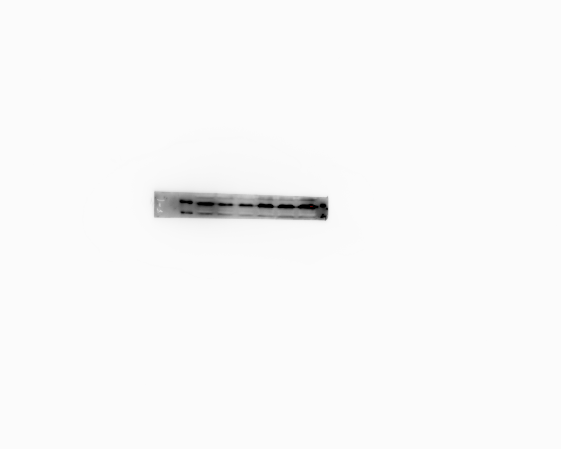

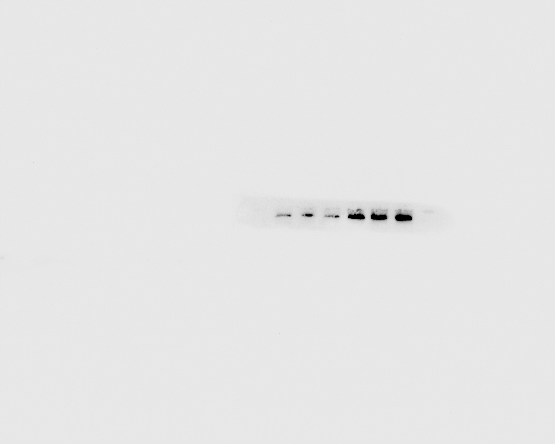


p16 GAPDH


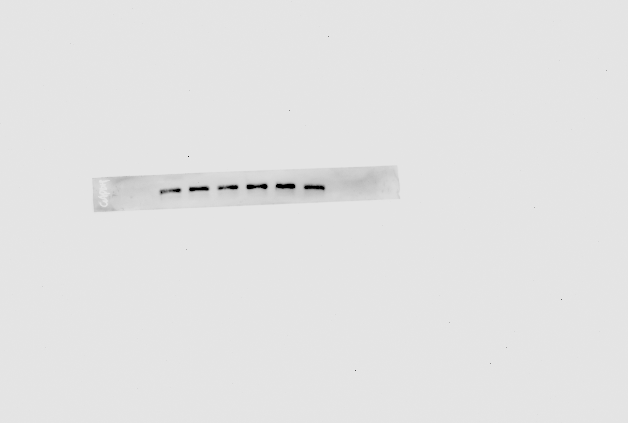

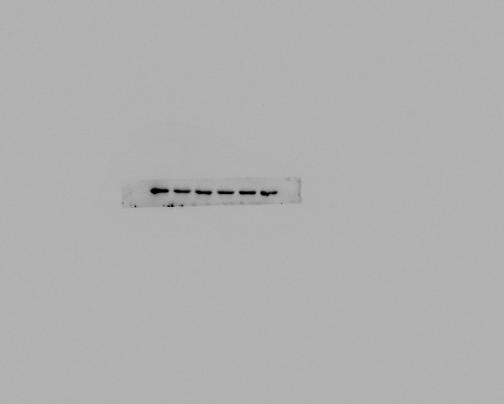


Panel g:

LC3 GAPDH


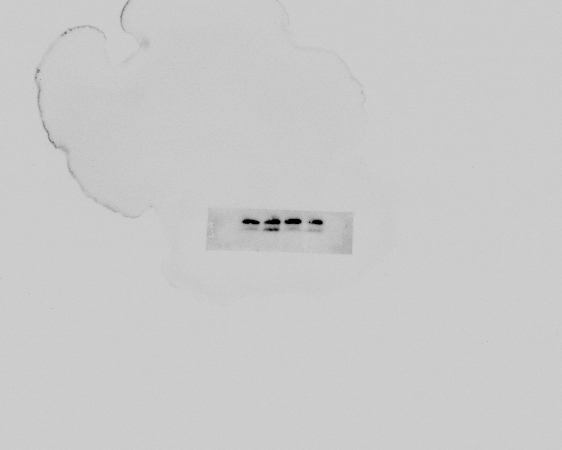

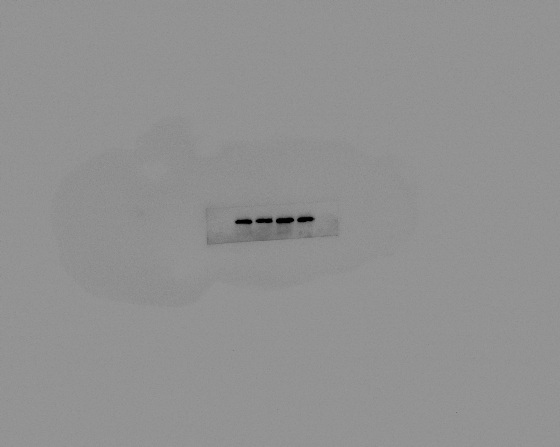


Panel h:

p62 GAPDH


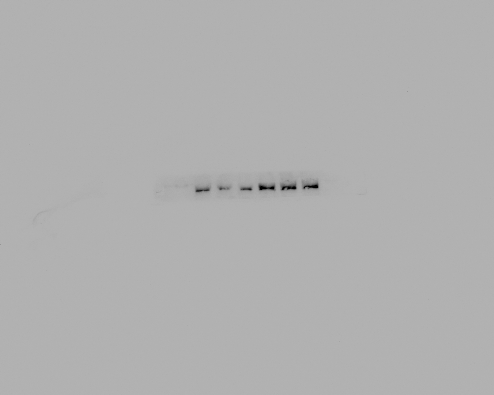

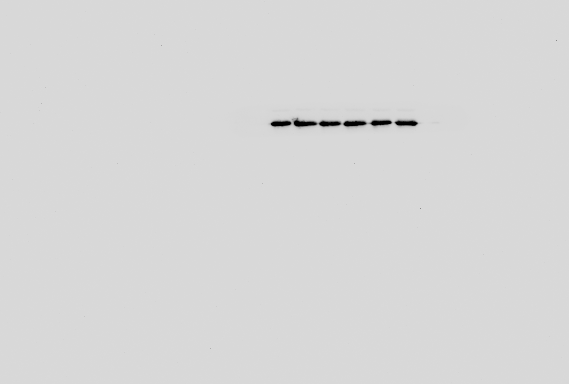


Panel k:

p53 p21


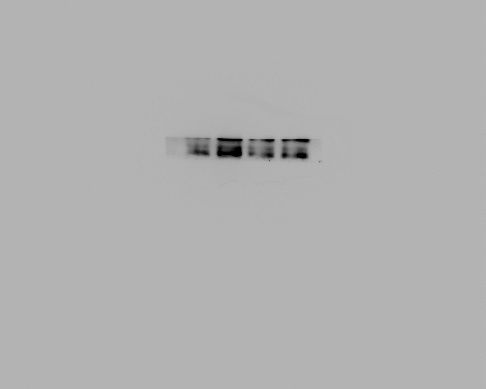

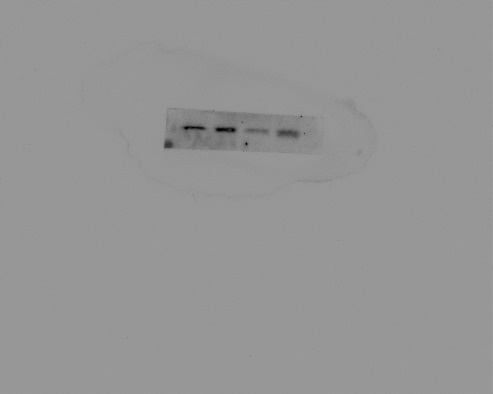


P16 GAPDH


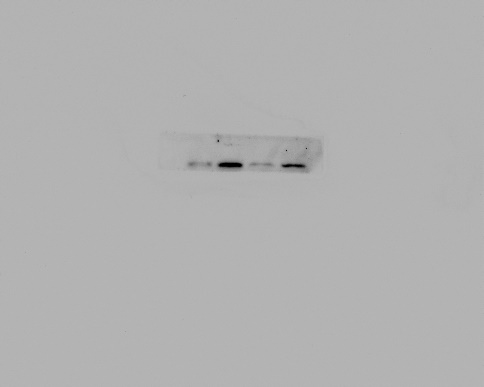

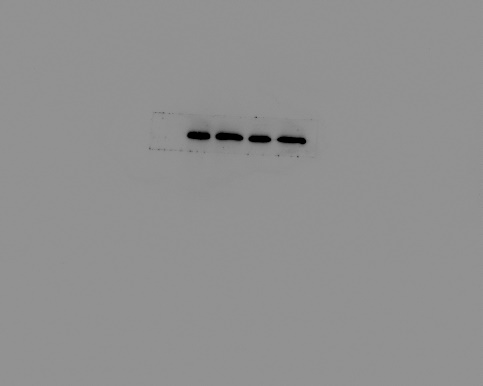


**Figure 3**

Panel b:

TFEB GAPDH


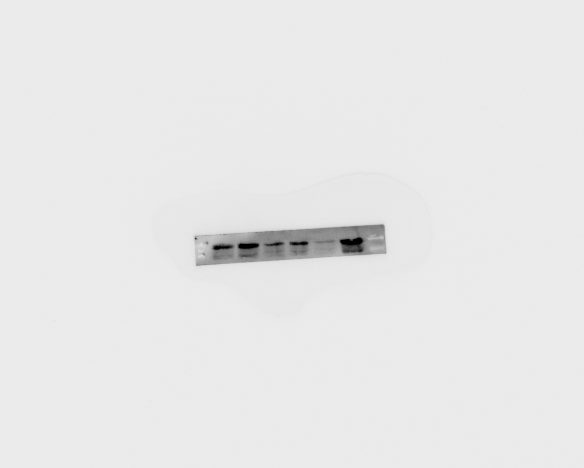

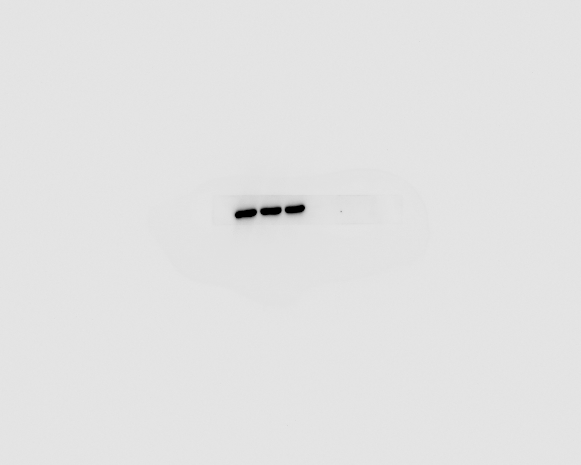


H3 p-TFEB


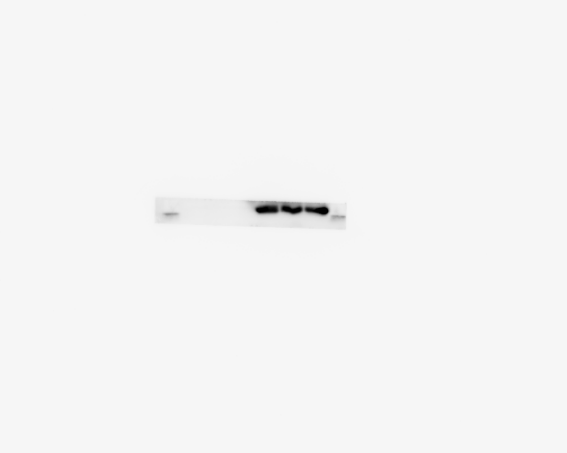

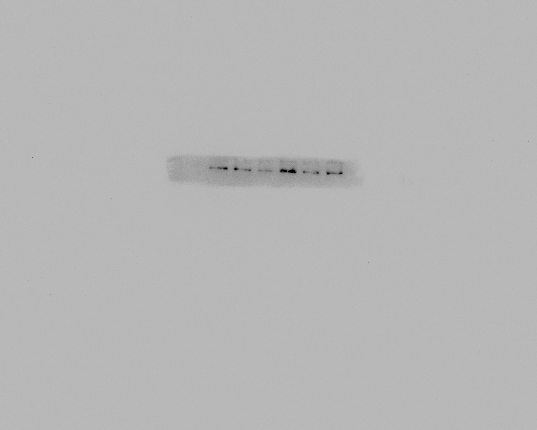


Panel g:

p53 p21


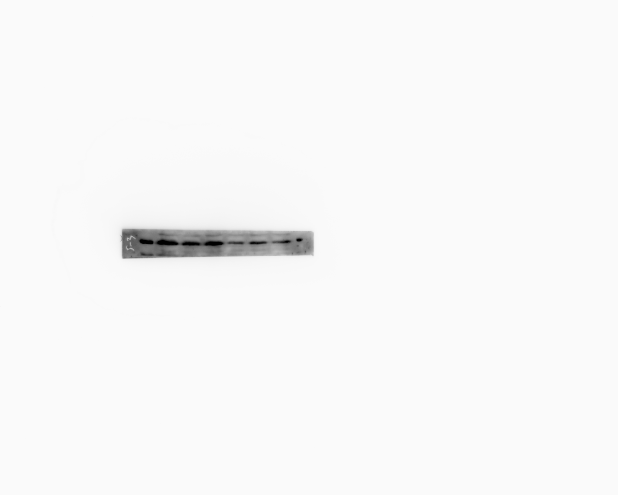

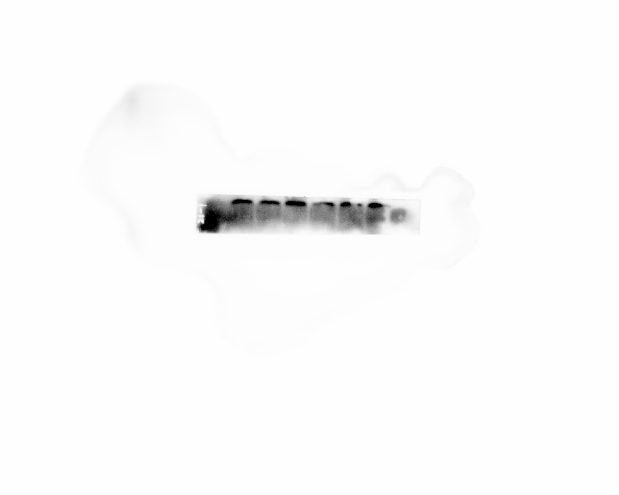


p16 GAPDH


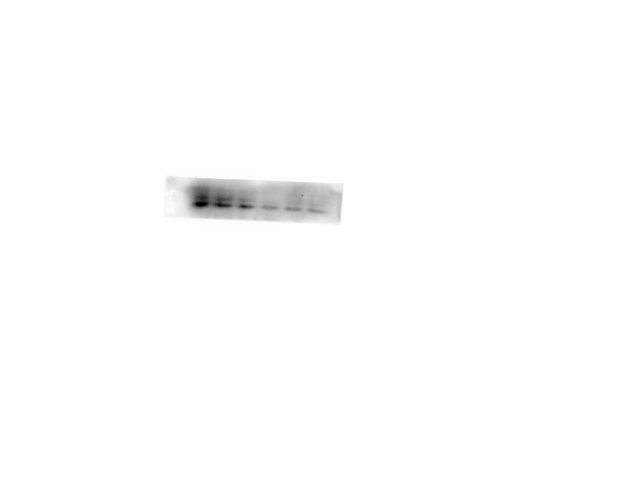

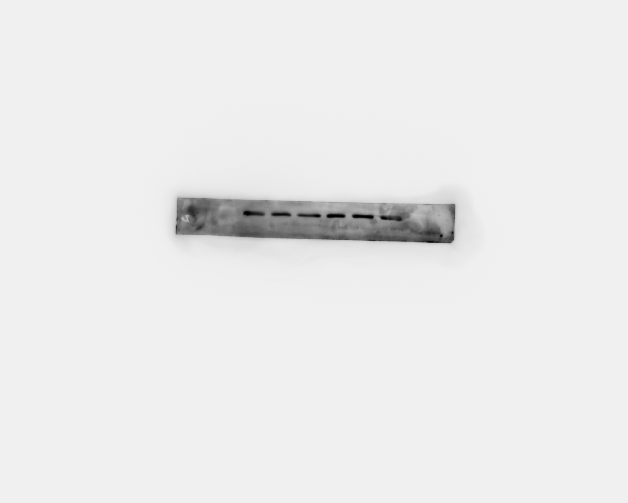


Panel i:

LC3 GAPDH


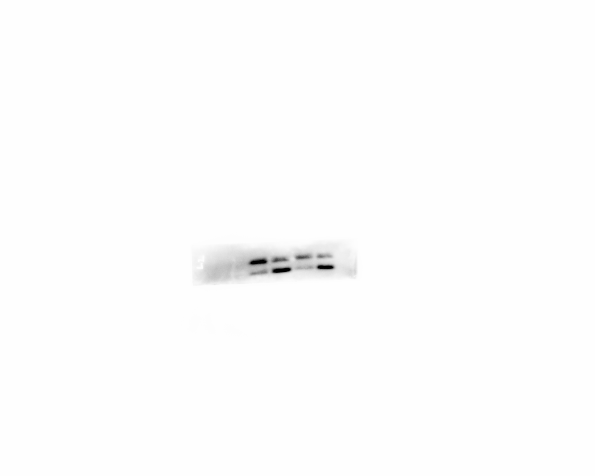

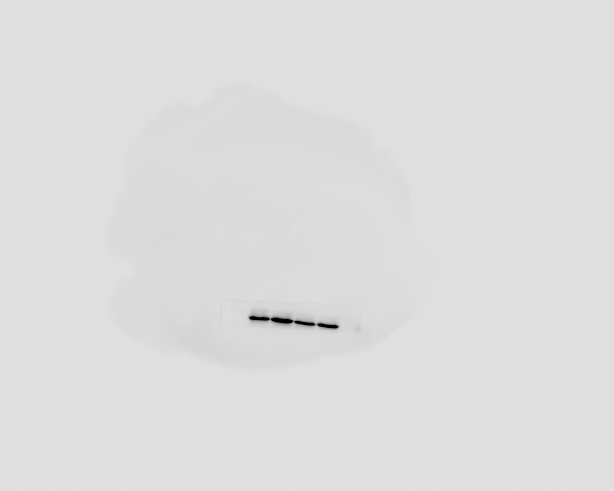


Panel j:

p62 GAPDH


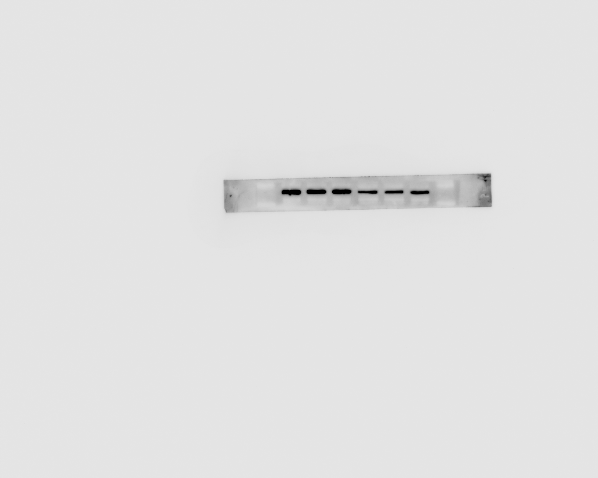

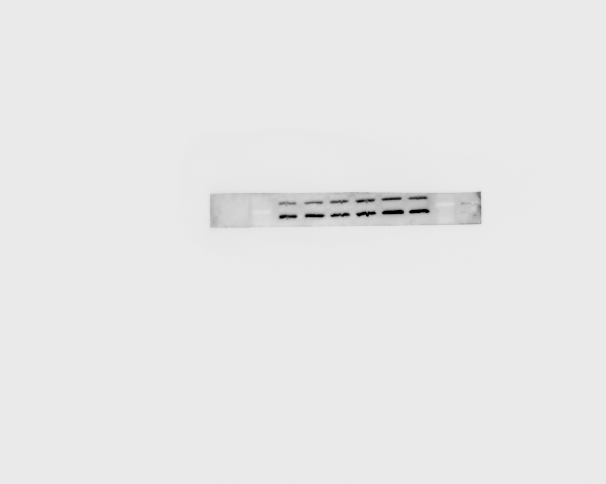


**Figure 4**

Panel c:

Flag-IP His-IP


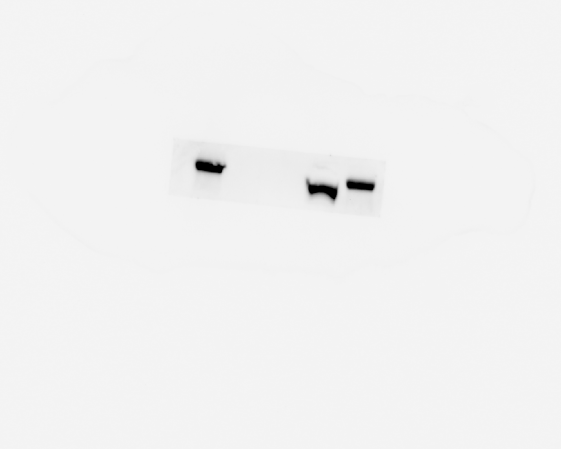

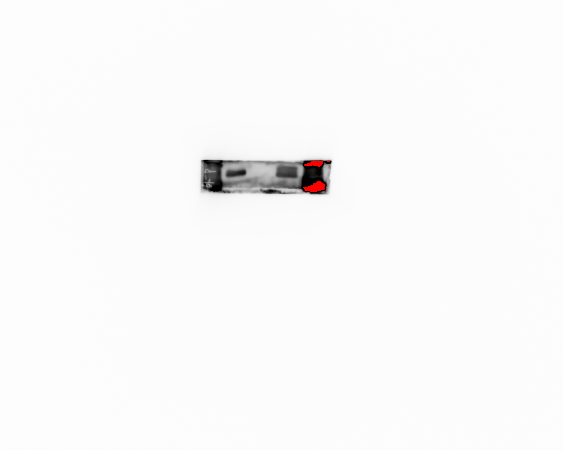


Flag-input His-input


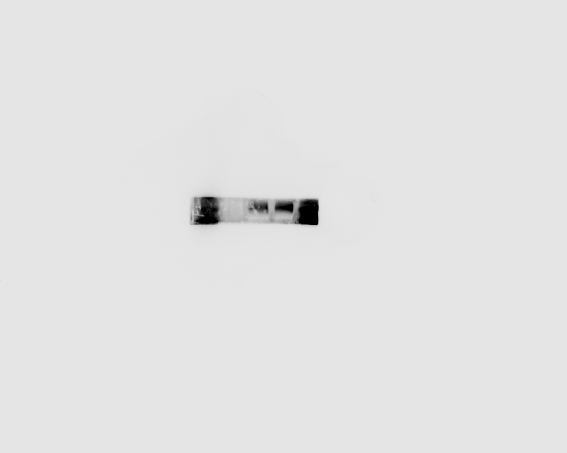

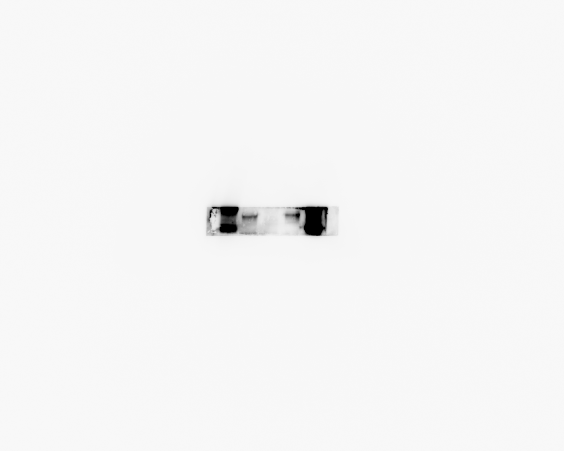


Panel d:

Flag-IP His-IP


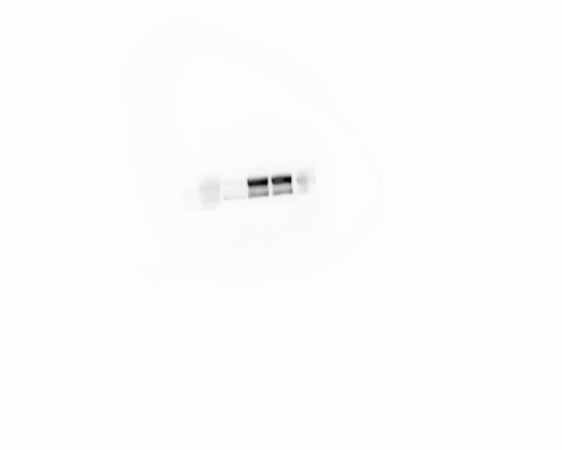

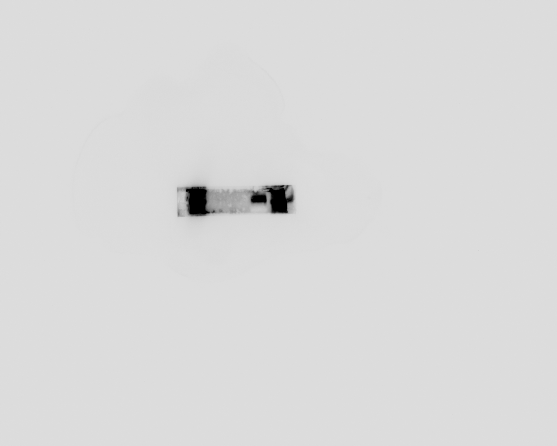


Flag-input His-input


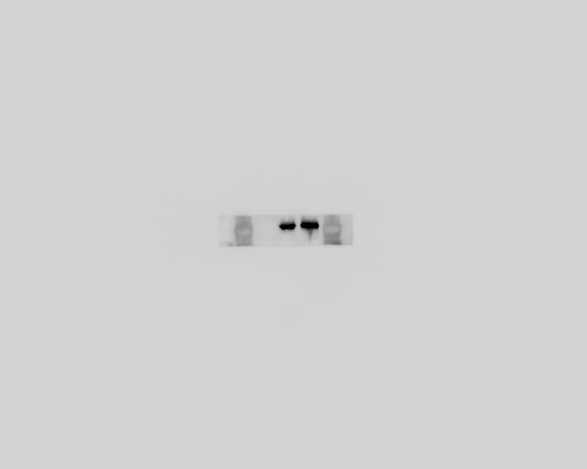

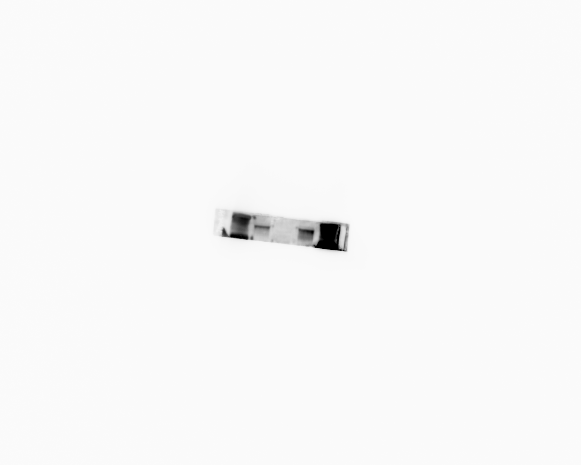


Panel e:

PPP1CA TFEB


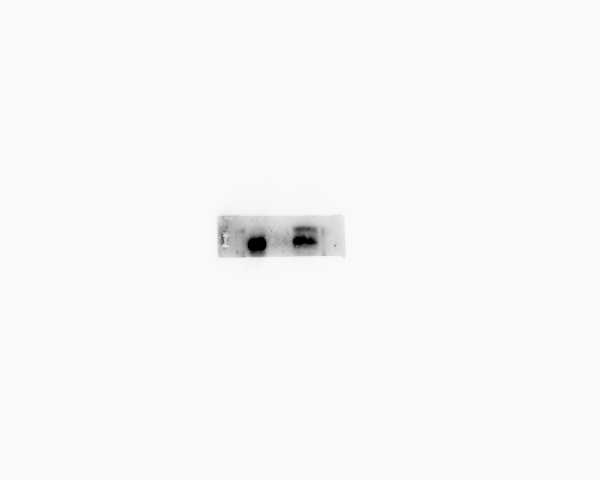

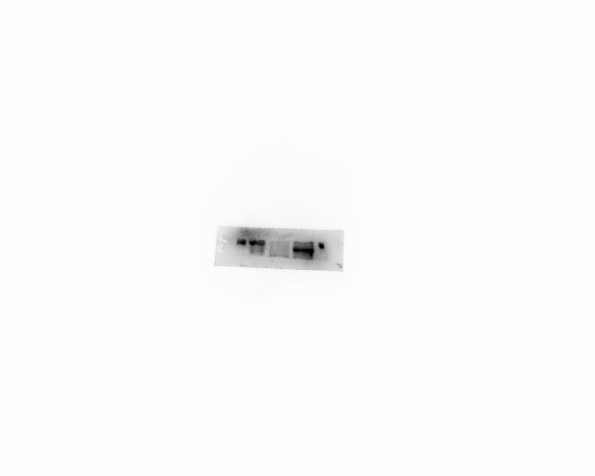
 Panel f:

PPP1CA TFEB


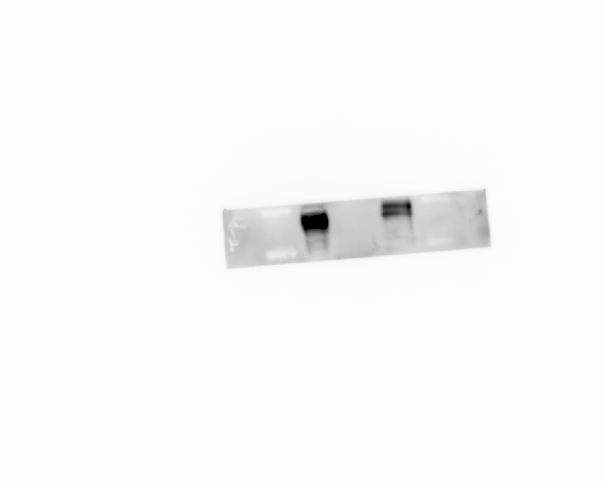

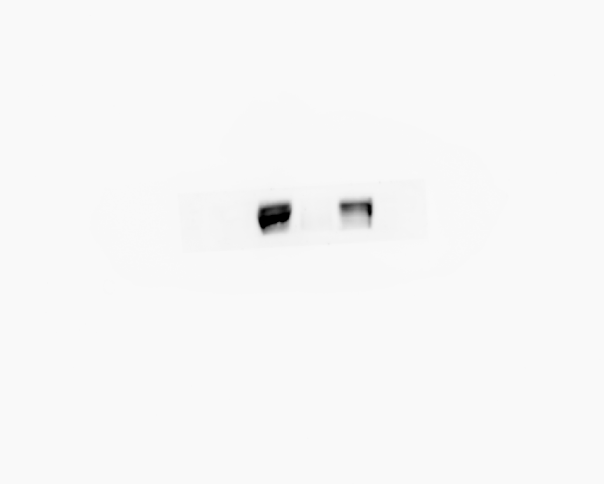


Panel g:

TFEB GAPDH


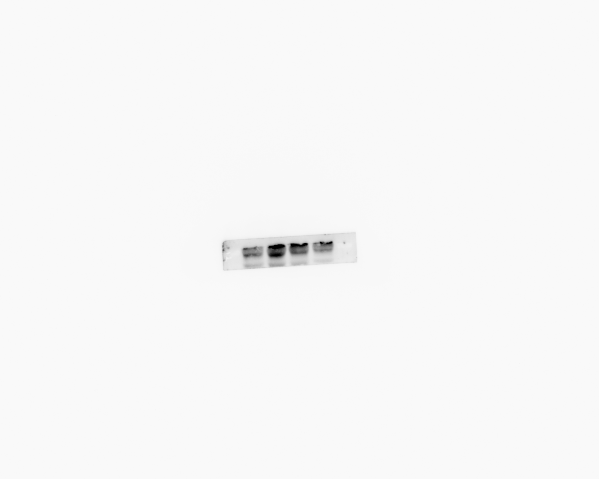

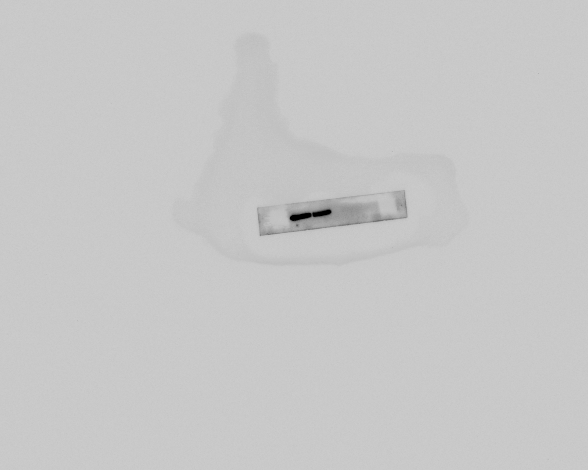


H3 p-TFEB


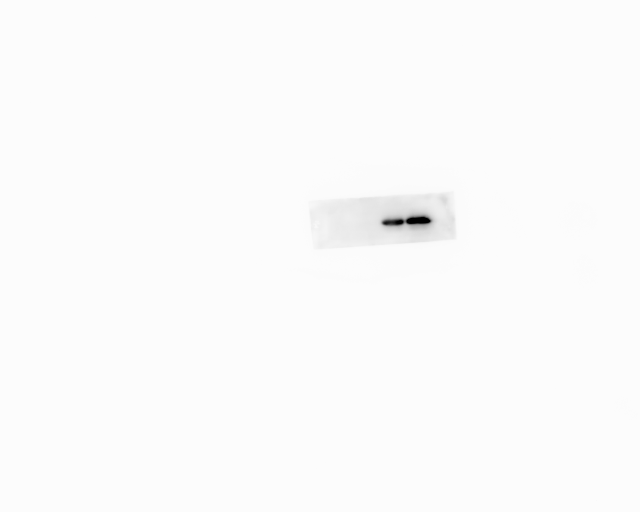

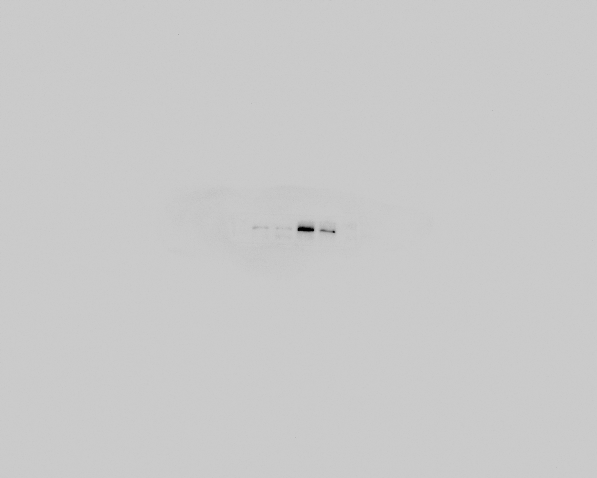


Panel h:

TFEB p-TFEB


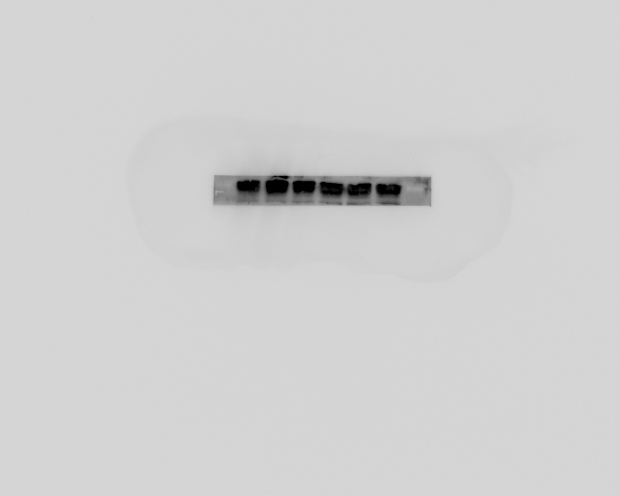

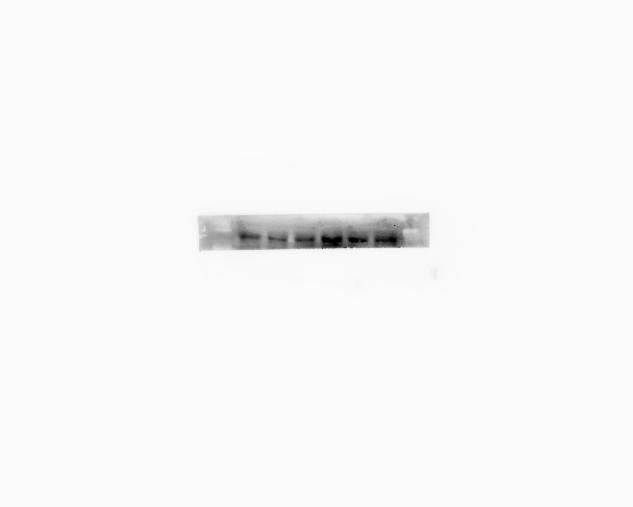


GAPDH


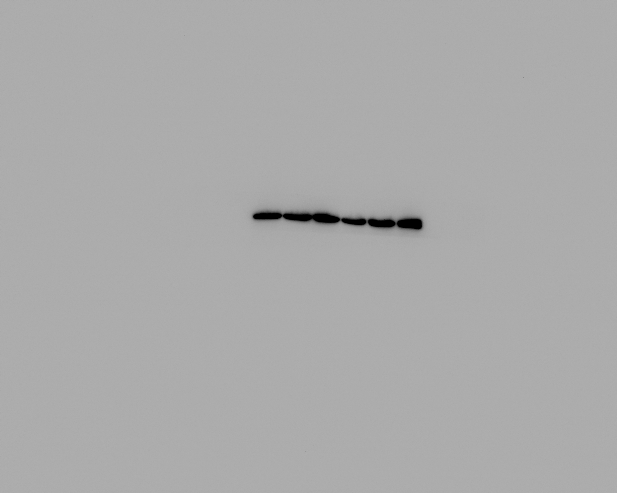


Panel k:

PPP1CA-ip Mono-methy


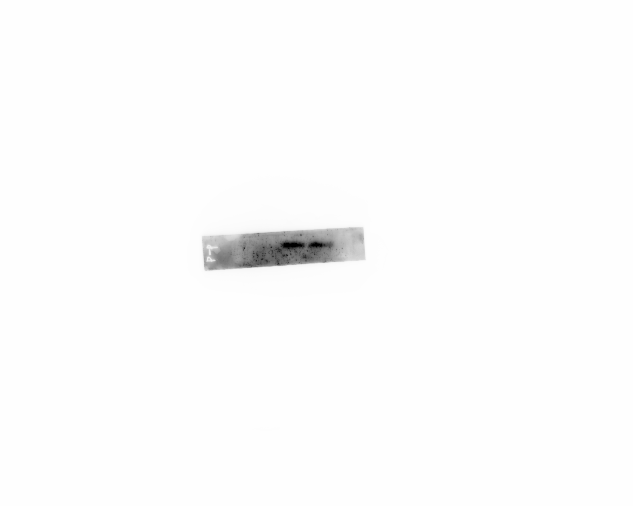

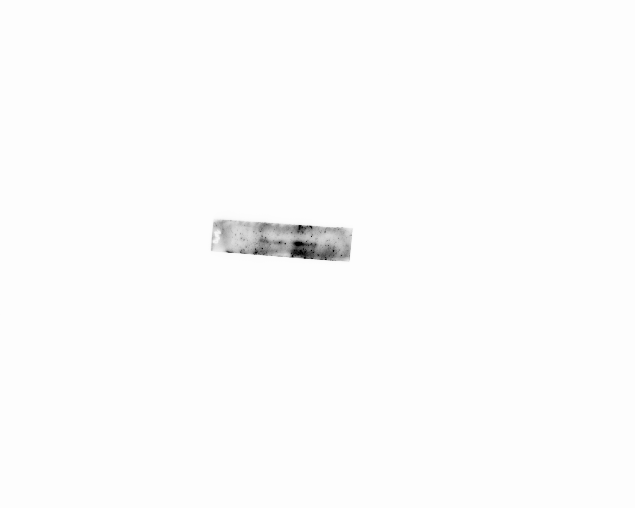


PPP1CA-input ACTIN-input


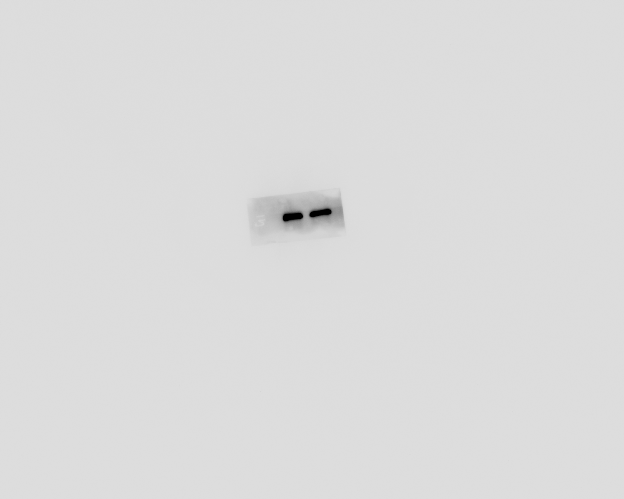

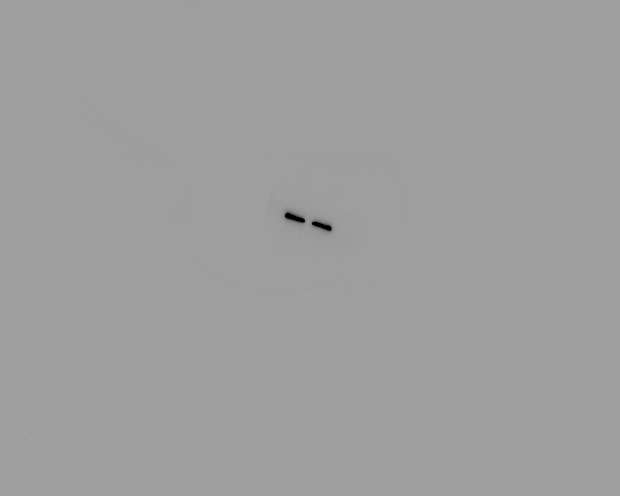


Panel l:

PPP1CA-ip Mono-methy


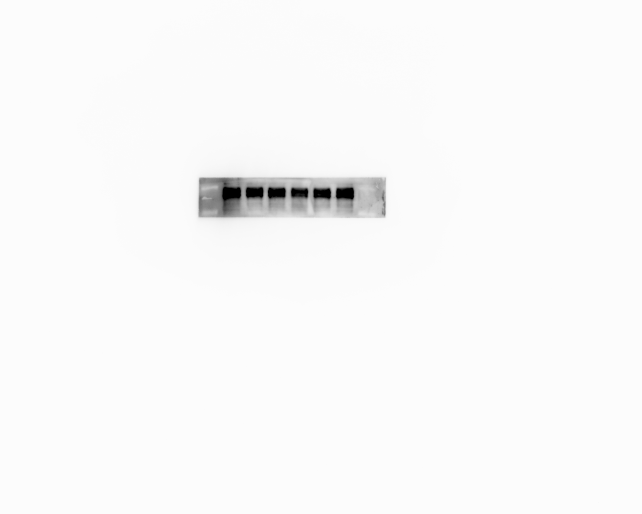

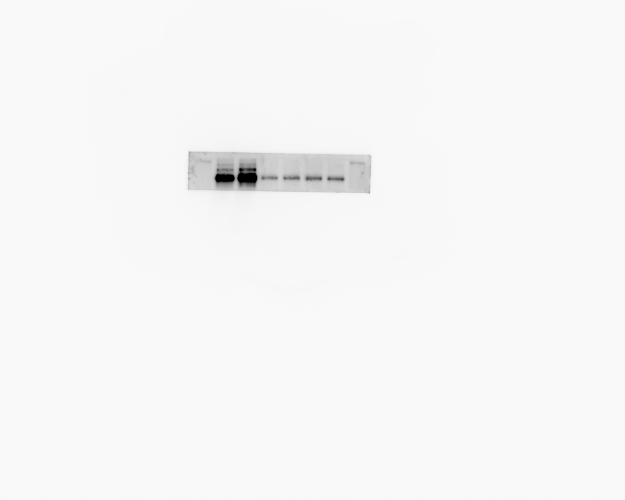
 PPP1CA-input ACTIN-input


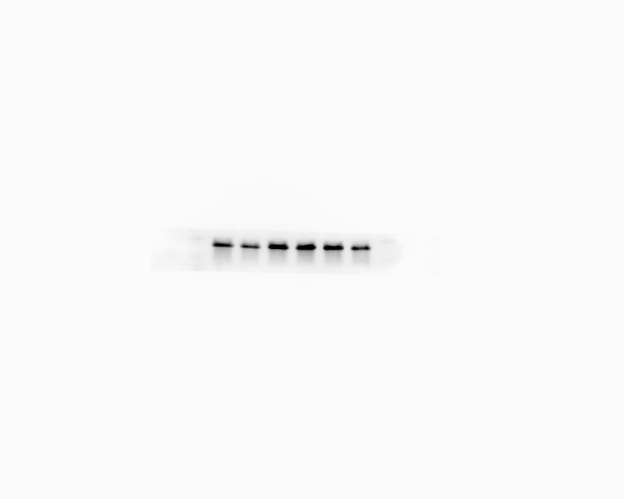

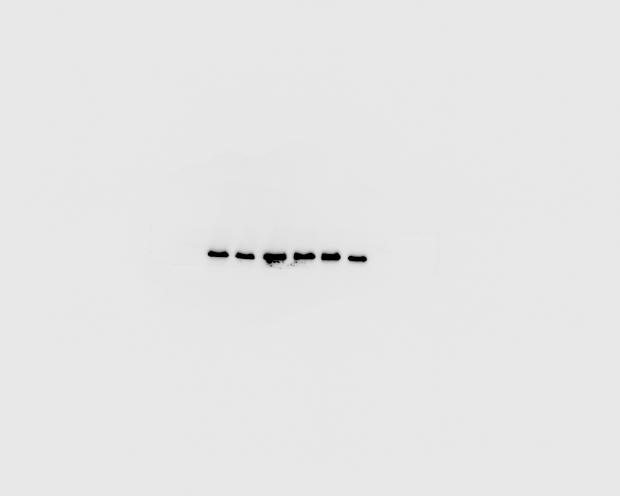


Panel n:

PPP1CA-ip TFEB-ip


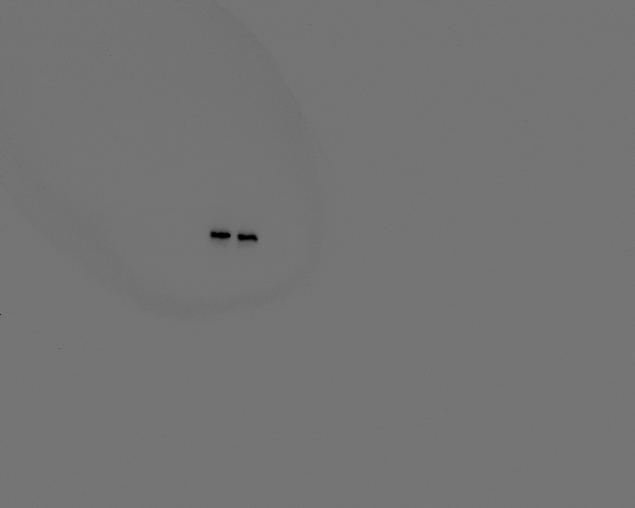

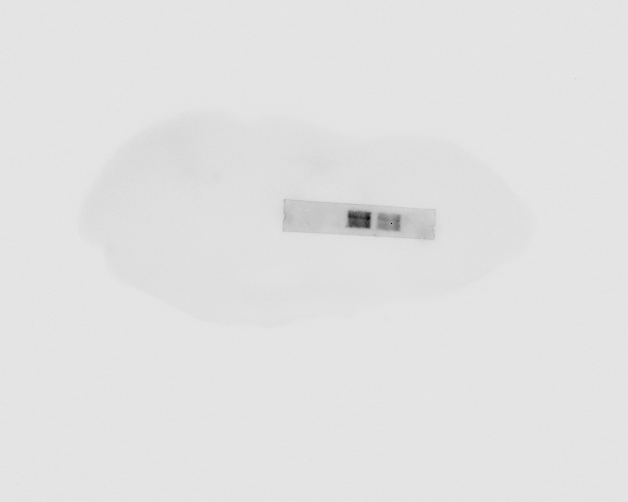


PPP1CA-input TFEB-input


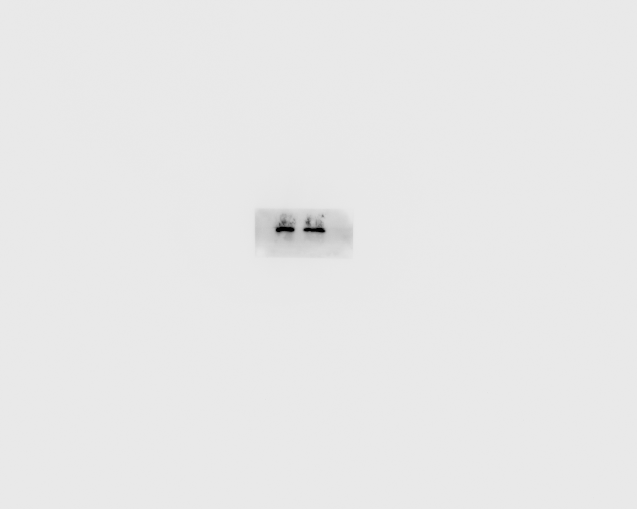

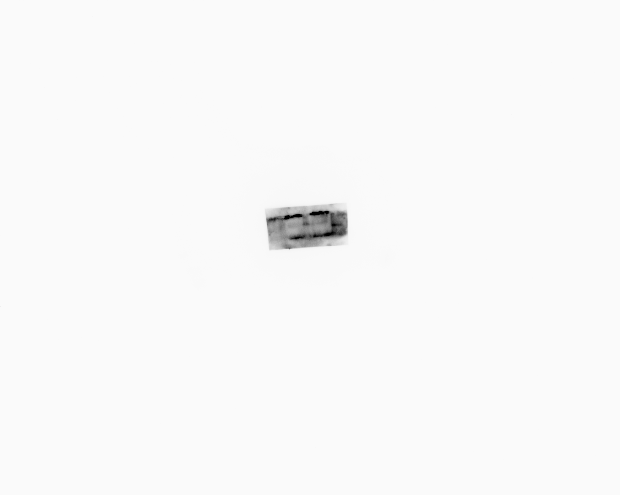


ACTIN-input


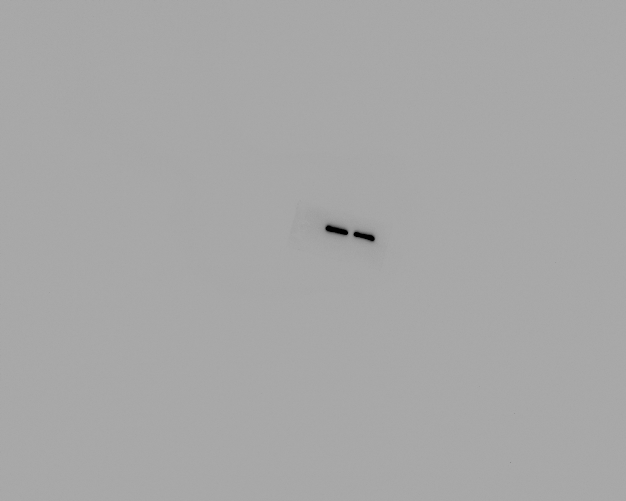


Panel o:

Flag-ip His-ip


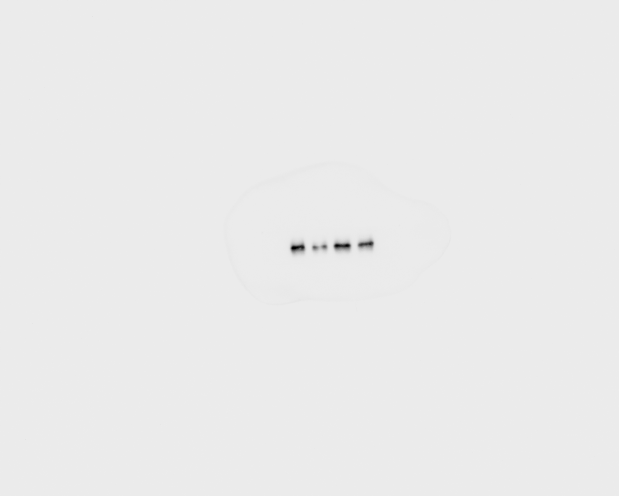

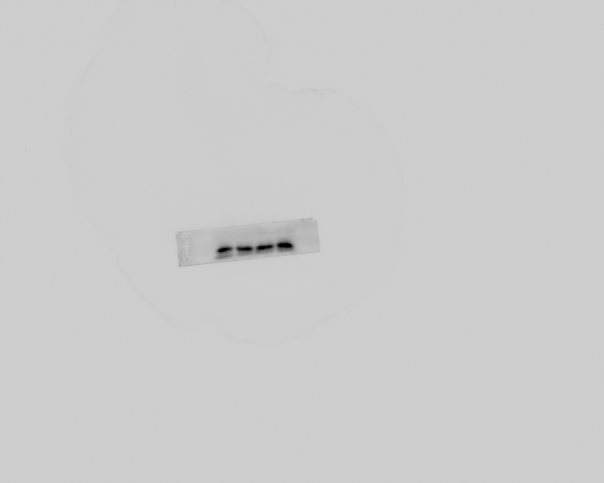


Flag-input His-input


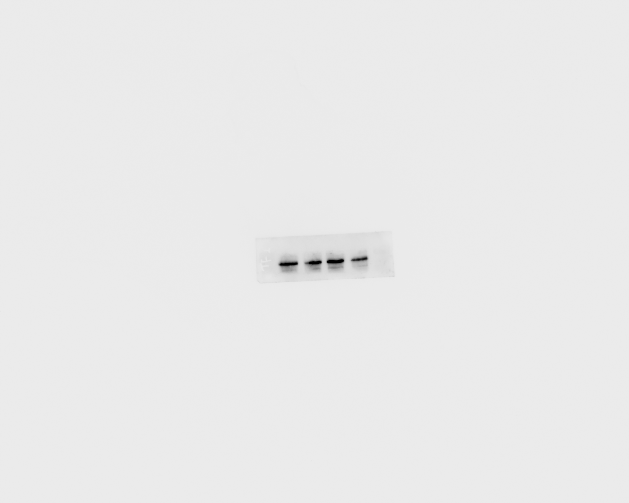

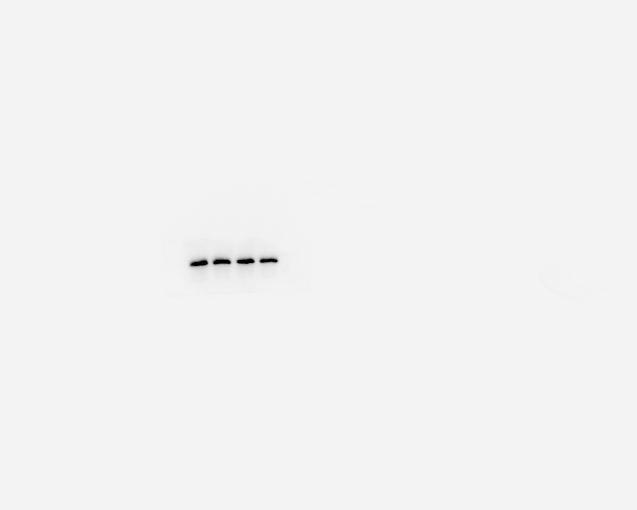


ACTIN-input


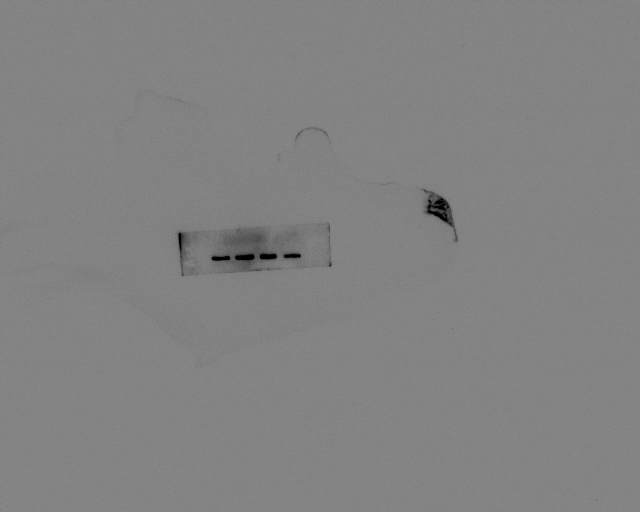


Panel p:

Flag-ip His-ip


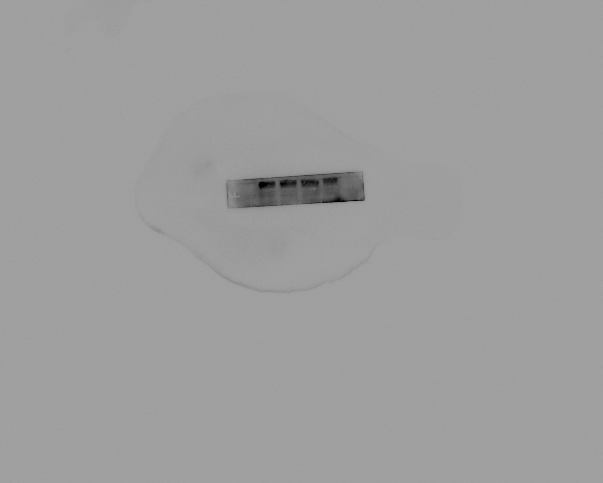

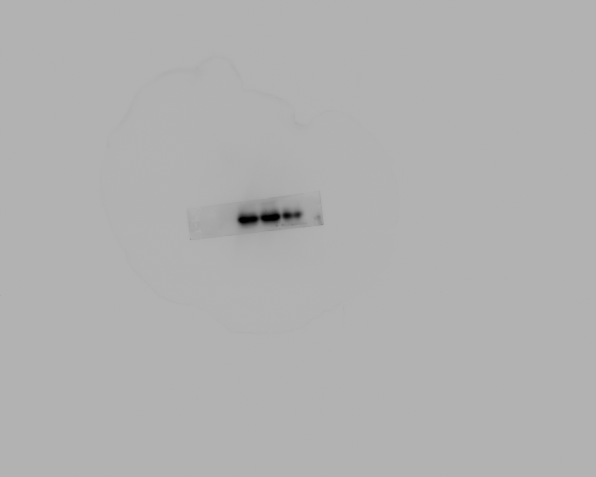


Flag-input His-input


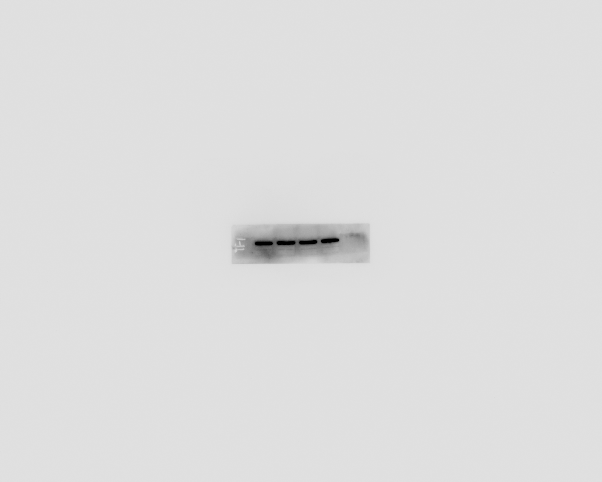

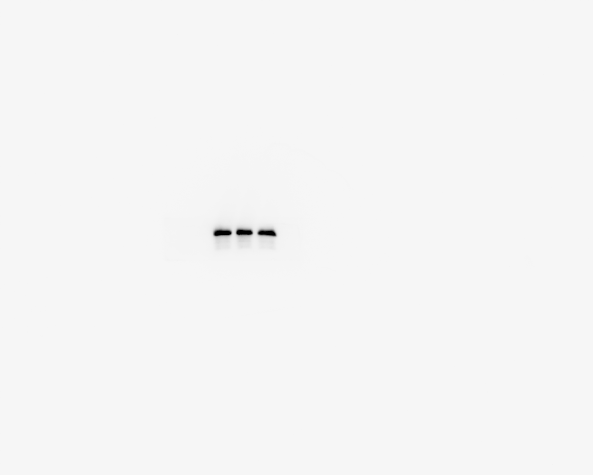


ACTIN-input


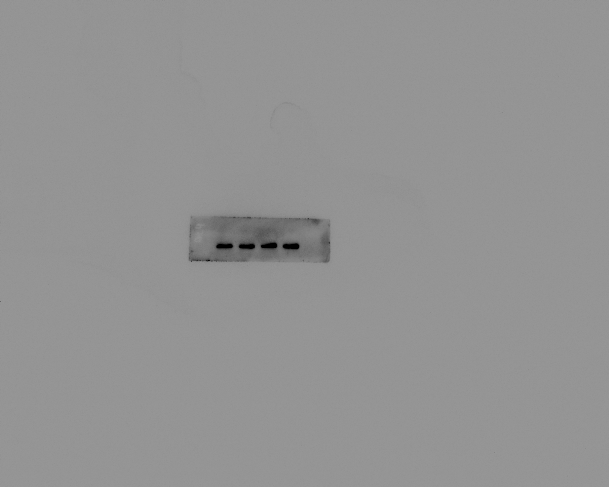


**Figure 5**

Panel d:

p53 p21


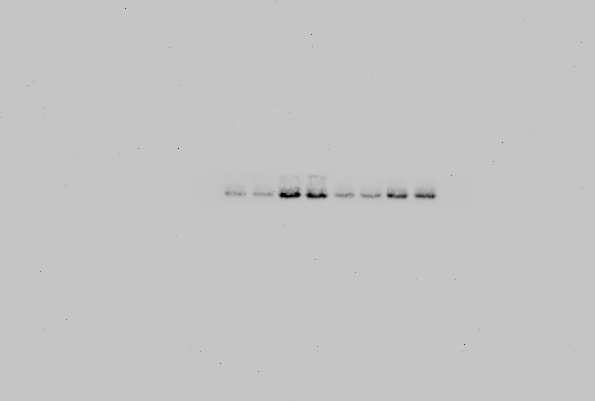

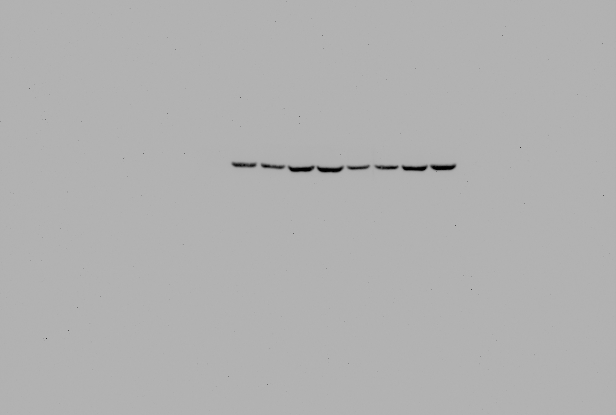


p16 GAPDH


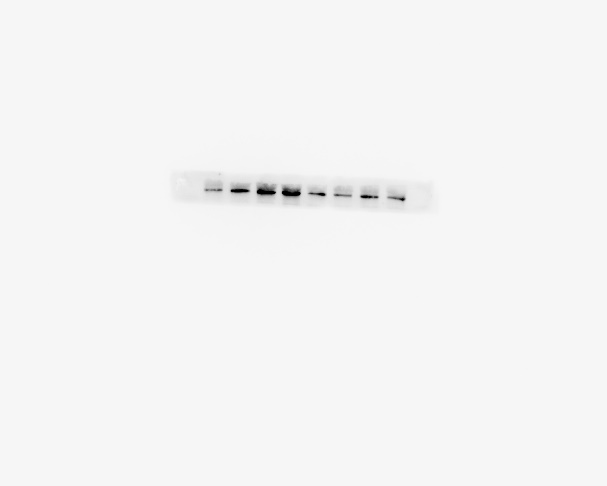

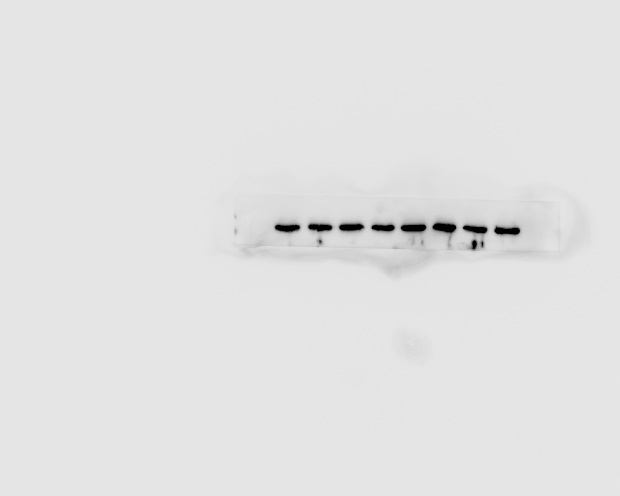


**Figure 6**

Panel b:

Gst-ip His-ip


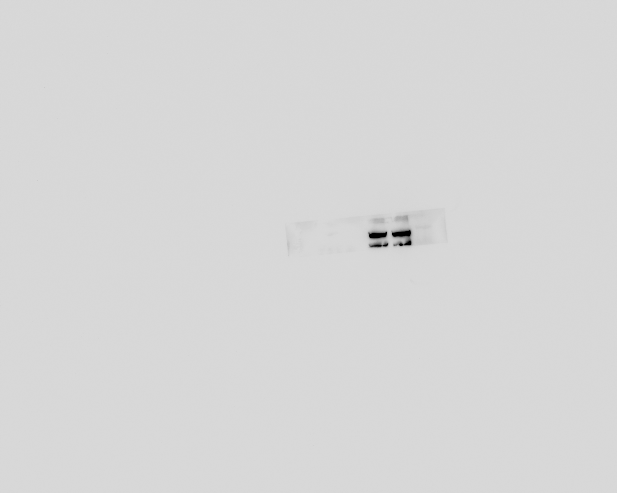

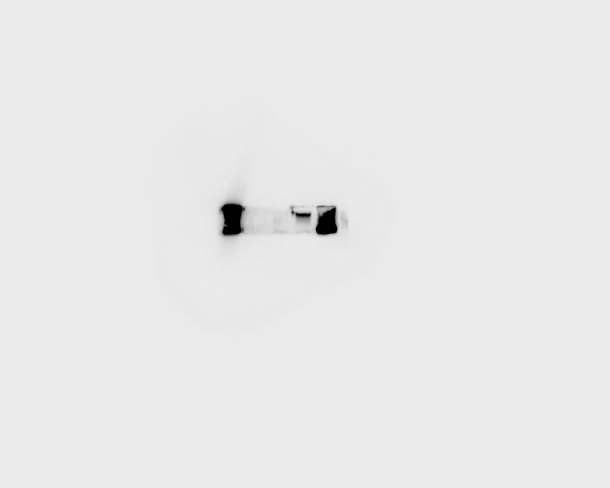


Gst-input His-input


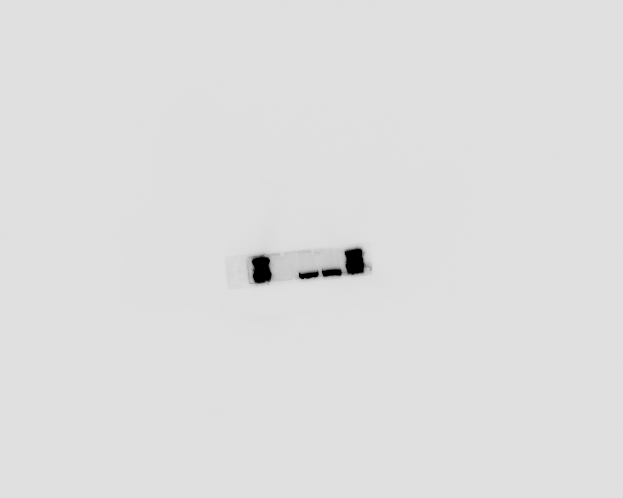


Panel c:

Gst-ip His-ip

Gst-input His-input

Panel d:

PPP1CA PPP1R9B

Panel d:

PPP1CA PPP1R9B

Panel e:

Gst-ip Flag-ip

Gst-input Flag-input

Panel f:

Gst-ip Flag-ip

Gst-input Flag-input

Panel g:

TFEB PPP1R9B

Panel g:

TFEB PPP1R9B

Panel h:

PPP1CA-input TFEB-input

PPP1R9B TFEB

PPP1R9B TFEB

Panel i:

TFEB GAPDH

H3 p-TFEB

Panel k:

TFEB p-TFEB

GAPDH

Panel m:

PPP1R9B-ip PPP1CA-ip

PPP1R9B-input PPP1CA-input

ACTIN-input

Panel n:

Gst-ip His-ip

Gst-input His-input

ACTIN-input

Panel o:

Gst-ip His-ip

Gst-input His-input

ACTIN-input

**Figure 7**

Panel b:

PP1 G9a

SETD1a SUV39H2

Panel c:

PP1 SUV39H2

Panel d:

PP1 SUV39H2

Panel e:

Mbp-ip His-ip

Mbp-input His-input

Panel f:

Mbp-ip His-ip

Mbp-input His-input

Panel g:

His-ip methyl-ip

His-input SUV39H2-input

ACTIN-input

Panel h:

His-ip methyl-ip

His-input ACTIN-input

Panel i:

PPP1CA-ip methyl-ip

PPP1CA-input ACTIN-input

Panel j:

PPP1CA-ip methyl-ip

PPP1CA-input SUV39H2-input

ACTIN-input

Panel k:

PPP1CA-ip TFEB-ip

PPP1R9B-ip methyl-ip

PPP1R9B-input TFEB-input

PPP1CA-input ACTIN-input

Panel l:

PPP1CA-ip TFEB-ip

PPP1R9B-ip methyl-ip

PPP1R9B-input TFEB-input

PPP1CA-input ACTIN-input

Panel n:

TFEB GAPDH

H3 p-TFEB

Panel m:

p-TFEB TFEB

GAPDH

**Figure 8**

Panel c:

SUV39H2 GAPDH

Panel e:

SUV39H2 GAPDH

Panel h:

p53 p21

p16 GAPDH

Panel i:

LC3 GAPDH

Panel k:

p62 GAPDH

**Figure S2**

Panel b:

TFEB GAPDH

Panel g:

TFEB-ip 14-3-3-ip

TFEB-input 14-3-3-input

ACTIN-input

Panel h:

p-TFEB TFEB

GAPDH

Panel i:

TFEB GAPDH

**Figure S4**

Panel a:

PPP1CA ACTIN

Panel c:

TFEB-ip 14-3-3-ip

TFEB-input 14-3-3-input

ACTIN-input

Panel d:

p-S6K S6K

p-4EBP1 4EBP1

GAPDH

**Figure S5**

Panel a:

LC3 GAPDH

**Figure S6**

Panel a:

PPP1R9B GAPDH

**Figure S7**

Panel b:

SUV39H2 GAPDH

**Figure S8**

Panel g:
